# Secondary traumatic stress and psychological distress among responders to the 2023 Kahramanmaraş earthquakes: a systematic review and meta-analysis

**DOI:** 10.3389/fpubh.2026.1967067

**Published:** 2026-10-08

**Authors:** Metin Çınaroğlu, Eda Yılmazer

**Affiliations:** 1 Psychology Department, Faculty of Administrative, Business and Social Sciences, İstanbul Nişantaşı University, İstanbul, Türkiye; 2 Psychology Department, Faculty of Arts and Sciences, Haliç University, İstanbul, Türkiye

**Keywords:** compassion fatigue, disaster responders, earthquake, meta-analysis, search and rescue, secondary traumatic stress, Türkiye

## Abstract

**Background:**

The Kahramanmaraş earthquakes on February 6, 2023 prompted one of the largest disaster response operations on record. Responders faced repeated exposure to death and distress, and locally recruited personnel were often disaster-affected themselves. Existing syntheses of this event excluded responders.

**Methods:**

Following PRISMA 2020 (PROSPERO-registered), seven Turkish and English sources were searched for studies on psychological outcomes among responders to these earthquakes. The primary outcome was secondary traumatic stress. Outcomes were pooled in random-effects models (restricted maximum likelihood, Hartung–Knapp–Sidik–Jonkman adjustment); prevalence after Freeman–Tukey transformation. Personal exposure was a pre-specified moderator.

**Results:**

Twenty-one studies were included; 13 quantitative studies contributed data on 2,894 responders. The pooled Secondary Traumatic Stress Scale total across four studies was 44.37 (95% CI 32.25–56.50; *I*^2^ = 99.2%). Heterogeneity appeared partly attributable to personal exposure: personnel resident in or affected by the disaster zone scored 48.80 against 38.40 among deployed personnel, an imprecisely estimated difference of 10.40 points (95% CI 1.57–19.23), with τ^2^ falling from 46.96 to 8.22 among deployed personnel. Pooled prevalence of probable post-traumatic stress disorder across three studies was 14.5% (8.5–21.5). Within the largest sample, personal exposure predicted secondary traumatic stress in graded fashion: physical injury Hedges’ *g* = 0.99, home destruction *g* = 0.70, bereavement *g* = 0.41.

**Conclusion:**

Whether the responder was also a victim was the most consistent modifier of outcome. Its direction was consistent, but its magnitude was imprecise and possibly small. Task allocation and post-deployment monitoring should be organized around personal exposure rather than professional role alone.

**Systematic review registration:**

https://www.crd.york.ac.uk/PROSPERO/view/CRD420261289019, identifier CRD420261289019.

## Introduction

At 04:17 local time on February 6, 2023, an earthquake of moment magnitude 7.7 struck the Pazarcık district of Kahramanmaraş in southeastern Türkiye. A second earthquake of magnitude 7.6 struck the neighboring Elbistan district about 9 h later (1). The two ruptures devastated 11 provinces containing more than 13 million people and caused severe damage in northwestern Syria. More than 50,000 people died in Türkiye alone, and over half a million buildings were destroyed or severely damaged (1). The response was correspondingly large. The Ministry of Health reported 18,022 doctors and 111,699 health personnel working at the earthquake sites, together with 14,435 National Medical Rescue Team members (2). Official figures record 35,250 search-and-rescue personnel in the field (3), drawn from the Disaster and Emergency Management Presidency, gendarmerie and police search-and-rescue units, the Ministry of National Defence, ambulance services, municipal fire departments, the Turkish Red Crescent and non-governmental organizations. More than 13,000 emergency personnel and volunteers from 100 countries were deployed alongside them, including urban search-and-rescue teams from 83 countries (4, 5). Very large numbers of people were therefore placed, often at short notice and for poorly bounded periods, in an operational environment defined by structural collapse, confined-space extrication, mass fatality management and sustained contact with survivors in acute distress (6, 7).

Research on survivors does not characterize this workforce adequately, because responder exposure differs in kind and not only in degree. It is occupational rather than incidental. It repeats across a career rather than resolving after a single event. It is also shaped by organizational structures with no counterpart in direct victimization, including team composition, leadership, shift patterns, and rest provision, all of which are known to modify outcome substantially (8, 9). Responder exposure is further characterized by indirectness. Secondary traumatic stress describes the intrusion, avoidance, and arousal symptoms that arise from knowing about, or assisting with, the traumatization of another person (10). It is measured with instruments built on that premise, most prominently the Secondary Traumatic Stress Scale (11), which has been adapted into Turkish by Kâhil (12) and by Yıldırım et al. (13). Two related constructs recur in this literature. Compassion fatigue incorporates both secondary traumatic stress and occupational burnout (14). Burnout is conceived instead as a response to chronic occupational demand rather than to traumatic content (15).

Several exposure features recur across the responder literature as correlates of morbidity. These include contact with human remains, participation in body recovery, identification with the deceased, and the experience of having been unable to reach those who could not be saved (8, 16). A further complication applies with unusual force to the present event. The affected area was geographically extensive and densely populated, so a substantial proportion of responding personnel were themselves resident in the 11 provinces. Those personnel were simultaneously helpers and victims, sustaining bereavement, injury, and property loss while on duty (17, 18). This dual role has been described as generating a distinctive strain, in which the affect associated with providing help conflicts with the affect arising from personal victimization (8, 19).

The mental health of rescue workers has been synthesized internationally on several occasions. Martínez and Blanch (20) reported a meta-regression of worldwide post-traumatic stress disorder (PTSD) prevalence among rescue workers, and Arena et al. (21) a meta-analysis of global prevalence among active first responders. Both found substantial but widely varying estimates, with heterogeneity attributable to occupational group, instrument, and time since exposure. Tahernejad et al. (22) addressed post-traumatic stress specifically among medical workers involved in earthquake response. Historical Turkish evidence provides a useful anchor: (87) found probable PTSD in a quarter of 55 search-and-rescue workers two months after the 2003 Bingöl earthquake. These syntheses pool across disasters of markedly different type, magnitude, and organizational context, and none was positioned to incorporate evidence from the 2023 Kahramanmaraş earthquakes, which have since generated a rapidly accumulating and substantially Turkish-language literature (23, 24).

Event-specific evidence remains fragmented, and the reviews that exist do not cover this population. Çınaroğlu et al. (25) meta-analyzed post-traumatic stress, depression, and anxiety following these earthquakes but restricted eligibility to general adult survivor samples, identifying the exclusion of rescue and healthcare workers as an explicit limitation. Grossi et al. (8) reviewed protective factors for resilience and well-being among natural-hazard helpers, but narratively rather than meta-analytically, across all hazard types worldwide, with single-reviewer screening and no prospective registration. Sehlikoğlu et al. (26) reviewed earthquake and mental health of healthcare workers, but their search was conducted before the index earthquakes occurred. Most recently, Ruhm et al. (2) scoped 35 years of literature on post-earthquake burnout among doctors and nurses in Türkiye and Syria, with the specific aim of locating structured preventive and early-intervention models. That review differs from the present one in four respects: it is a scoping rather than a systematic review and performs no quantitative synthesis; it spans 1990–2024 rather than addressing the index event; it is restricted to doctors and nurses, excluding search-and-rescue personnel, firefighters, volunteers, and psychosocial support teams; and its organizing outcome is burnout and intervention architecture rather than secondary traumatic stress. No review has yet addressed the full responder spectrum of these earthquakes as a population in its own right, or pooled outcomes across it.

Primary studies have meanwhile appeared across that spectrum without being synthesized. They include work among firefighters (6), National Medical Rescue Team personnel (27), Turkish Red Crescent staff (28), search-and-rescue workers (29), mental health professionals (30, 31), psychosocial support teams (32, 33), social workers (34), volunteers (35–37), and disaster response workers (9, 23). Hospital-based health personnel in the affected provinces have received particular attention. Koca et al. (38) modelled psychological resilience, stress coping and traumatic stress among 315 hospital staff across the 11 declared provinces and found that resilience exerted a significant negative effect on traumatic stress symptoms. Taş et al. (39) compared health workers who had experienced the earthquake with those deployed from unaffected provinces, and Derya et al. (40) examined trauma level, compassion fatigue, secondary traumatic stress, and burnout in a single clinician sample.

This review therefore addressed four questions. First, what is the level of secondary traumatic stress in this workforce, and of the related outcomes of depression, anxiety, acute stress, burnout, compassion fatigue, and resilience? Second, what is the pooled prevalence of probable PTSD among personnel who responded to the February 6, 2023 earthquakes? Third, does personal earthquake exposure modify responder outcome, and if so with what magnitude? Fourth, what do responders themselves identify as having made the work bearable? This study expected secondary traumatic stress to be elevated in all sampled groups, and also expected pooled PTSD prevalence to fall within the range reported for first responders after other major earthquakes. In addition, personal earthquake exposure was expected to be associated with higher secondary traumatic stress in graded fashion, with magnitude ordered by severity of loss, both within samples and across them.

## Methods

### Protocol and registration

The protocol was registered with the International Prospective Register of Systematic Reviews (PROSPERO; CRD420261289019). The review is reported in accordance with the PRISMA 2020 statement (41), and the completed checklist is provided as Supplementary File S2. Outcomes that did not meet the pre-specified threshold for pooling are additionally reported in accordance with the Synthesis Without Meta-analysis guideline (42). Deviations from the registered protocol are reported at the end of this section.

### Eligibility criteria

Eligible participants were individuals aged 18 years or over who took part in the response to the index earthquakes in a professional or volunteer capacity. This encompassed urban search-and-rescue personnel, firefighters, Disaster and Emergency Management Presidency and National Medical Rescue Team staff and volunteers, Turkish Red Crescent personnel, military and gendarmerie units assigned to response duties, emergency medical and other health personnel deployed to or working within the affected provinces, psychosocial support and mental health teams, social workers, and unaffiliated volunteers. The eligible exposure was occupational or volunteer engagement in disaster response related to these earthquakes, whether by deployment into the affected zone from elsewhere or by the provision of services within it by personnel already resident there. Health personnel working within the affected provinces were retained rather than excluded as survivors, on two grounds: the delivery of clinical care to earthquake casualties constitutes a response role, and such personnel are consistently identified as an at-risk occupational group (22, 26). Personnel based outside the affected provinces who delivered clinical care to evacuated earthquake casualties were also treated as fulfilling a response role, and were coded as a distinct deployment category. Deployment status was coded for every study and treated as a moderator of substantive interest. Studies of the general population, of survivors not acting in a response role, and of individuals exposed only through media were excluded.

The primary outcome was secondary traumatic stress assessed with a validated instrument. Secondary outcomes were post-traumatic stress symptoms, earthquake-specific trauma level, depression, anxiety, acute stress, burnout, compassion fatigue and satisfaction, psychological resilience, and post-traumatic growth. Quantitative observational designs, and interventional designs reporting baseline outcome data, were eligible for quantitative synthesis. Qualitative and mixed-method studies were eligible for thematic synthesis only. Peer-reviewed articles and postgraduate theses published in Turkish or English from February 6, 2023 to the final search date were eligible. Case reports, commentaries, editorials, and conference abstracts without data were excluded. Studies reporting only investigator-designed symptom items without established psychometric properties were excluded from quantitative synthesis but retained for narrative description.

### Information sources and search strategy

Seven sources were searched: Web of Science Core Collection, PubMed, Scopus, PsycINFO, TR Dizin, DergiPark, and the Council of Higher Education National Thesis Centre (YÖK Ulusal Tez Merkezi). The inclusion of the three Turkish sources is material. TR Dizin and DergiPark index the majority of Turkish-language health and social science journals in which research on this event has appeared and which are not indexed internationally, and the National Thesis Centre is the principal route to postgraduate gray literature on the event. Searches combined three concept blocks covering responder population, index event, and psychological outcome, with parallel strategies constructed in English and in Turkish. They were supplemented by backward citation chasing through the reference lists of all included reports and relevant reviews, and by forward citation tracking of all included studies. Corresponding authors were contacted where distributional parameters necessary for synthesis were absent. Complete strategies, with database-specific syntax, execution dates and record yields, are provided as Supplementary File S1.

### Study selection, data extraction, and verification

Records were de-duplicated and screened by title and abstract independently and in duplicate by the two authors. Full texts of all potentially eligible records were assessed independently against the eligibility criteria by the same two reviewers. Disagreements were resolved by discussion, and inter-rater agreement was quantified using Cohen’s κ. Data were extracted onto a piloted form by one author and independently verified by the second. Extracted fields were bibliographic details, design, sampling method, responder type, deployment status, interval since the earthquakes, sample size, mean age, proportion female, instrument and Turkish validation reference with any applied cut-off, internal consistency as reported, means and standard deviations, and the number and proportion above cut-off. Where studies reported subgroup contrasts by personal earthquake exposure, subgroup means, standard deviations, and cell sizes were extracted separately. The extraction form is provided as Supplementary File S3.

Every extracted value was verified against the source article’s own results tables before entry. Four checks were applied. Reported subgroup counts were required to sum to the stated total. Reported percentages were required to reproduce from their implied numerator and denominator. Reported confidence intervals were required to reconstruct from the point estimate and standard error. Values appearing in abstracts or discussions were required to agree with the corresponding results table. Where a discrepancy was identified and the primary table was internally coherent, the table value was used, the discrepancy was recorded, and the finding was incorporated into the statistical analysis domain of the risk-of-bias assessment. Where a discrepancy could not be resolved against a coherent table, the affected value was excluded from synthesis. This procedure was pre-specified and proved consequential across the evidence base, as reported below and in full in Supplementary File S4.

Two transformations were applied to make otherwise incomparable values poolable, and both are identified as such wherever they appear. Where a study reported scale scores as item means rather than totals, the total was reconstructed by multiplication by the number of items. Where a study reported medians with interquartile ranges only, means and standard deviations were estimated using the method of Wan et al. (43). Where a study reported subgrou*p* values but no overall value, the total was reconstructed by weighting; the reconstruction was accepted only where independent subgroup breakdowns converged.

### Risk of bias

Risk of bias was assessed independently and in duplicate using the Joanna Briggs Institute critical appraisal checklists appropriate to each design (44), covering the clarity of inclusion criteria, the description of subjects and setting, the validity and reliability of exposure and outcome measurement, the identification and handling of confounding, and the appropriateness of the statistical analysis. Studies were not excluded on the basis of appraisal score. Domain-level assessments for every included study are reported in Supplementary File S4.

### Effect measures and synthesis

For continuously scored outcomes reported on a common instrument, means with standard deviations were reported on the raw metric of the instrument and pooled where three or more studies contributed. Where studies reported a contrast between exposure groups, standardized mean differences were computed as Hedges’ *g* with the small-sample correction (45). For dichotomous outcomes defined by an instrument cut-off, proportions were computed with Wilson score confidence intervals (46).

Meta-analysis was pre-specified to require a minimum of three studies reporting an outcome on a comparable metric. Three outcomes met this threshold: secondary traumatic stress on the Secondary Traumatic Stress Scale, compassion fatigue on the Compassion Fatigue Short Scale, and probable PTSD prevalence. Continuous outcomes were pooled on the raw instrument metric in random-effects models, with between-study variance estimated by restricted maximum likelihood and confidence intervals derived using the Hartung–Knapp adjustment (47). For prevalence, because contributing studies used different instruments and different validated thresholds, proportions were stabilized using the Freeman–Tukey double arcsine transformation (48) with variance 1/(*n* + 0.5), pooled on the transformed scale and back-transformed using the harmonic mean of the sample sizes (49, 50). Heterogeneity was quantified with τ^2^, Cochran’s *Q* and *I*^2^ (51), and a 95% prediction interval was reported alongside each pooled estimate (52). Robustness was examined by leave-one-out estimation and by comparison with a fixed-effect model. Pre-specified moderators were responder type, deployment status, interval since the earthquakes, proportion female, and instrument adaptation. Deployment status and instrument adaptation could be examined by subgroup analysis for the primary outcome; the remainder could not. Small-study effects were to be assessed by funnel plot and Egger’s regression where at least 10 studies contributed to an outcome, and no outcome reached that threshold. Outcomes reported by fewer than three studies were synthesized narratively following Campbell et al. (42).

Analyses were conducted in Python version 3.11 (Python Software Foundation, Wilmington, DE, USA) using the NumPy (version 1.26) and SciPy (version 1.11) libraries. The analysis code and the extraction dataset are provided as Supplementary File S6, and all reported values can be reproduced from them.

### Deviations from the registered protocol

Three deviations are reported. First, the search was updated after peer review and expanded to seven sources; the amended execution dates and yields are recorded in Supplementary File S1. Second, a third deployment category was added during extraction for personnel based outside the affected provinces who treated evacuated casualties, a configuration the protocol had not anticipated. Third, the transformations described in the preceding subsection were applied to render item-mean, median, and subgroup-only reporting comparable; these were not pre-specified. No other departure from the registered protocol was made.

## Results

### Study selection

The flow of records through identification, screening, and inclusion is presented in Figure 1. Searches of the seven sources returned 1,247 records: 186 from Web of Science, 143 from PubMed, 214 from Scopus, 97 from PsycINFO, 258 from TR Dizin, 291 from DergiPark, and 58 from the National Thesis Centre. After removal of 389 duplicates, 858 records were screened by title and abstract and 824 were excluded. Of the 34 reports sought for retrieval, three could not be obtained, and these are listed in Supplementary File S5. Of the 31 reports assessed at full text, 10 were excluded and 21 met the eligibility criteria. Inter-rater agreement at full-text assessment was substantial (κ = 0.84). Thirteen included studies were quantitative and contributed data on 2,894 responders: 12 entered quantitative synthesis, of which 9 contributed to at least one meta-analysis, and one was retained for narrative description only because its outcomes were assessed with investigator-designed items. The remaining eight reports were qualitative or mixed-method and contributed data on 810 responders to the thematic synthesis. Reasons for exclusion at full text are given in Supplementary File S5.

**Figure 1:**
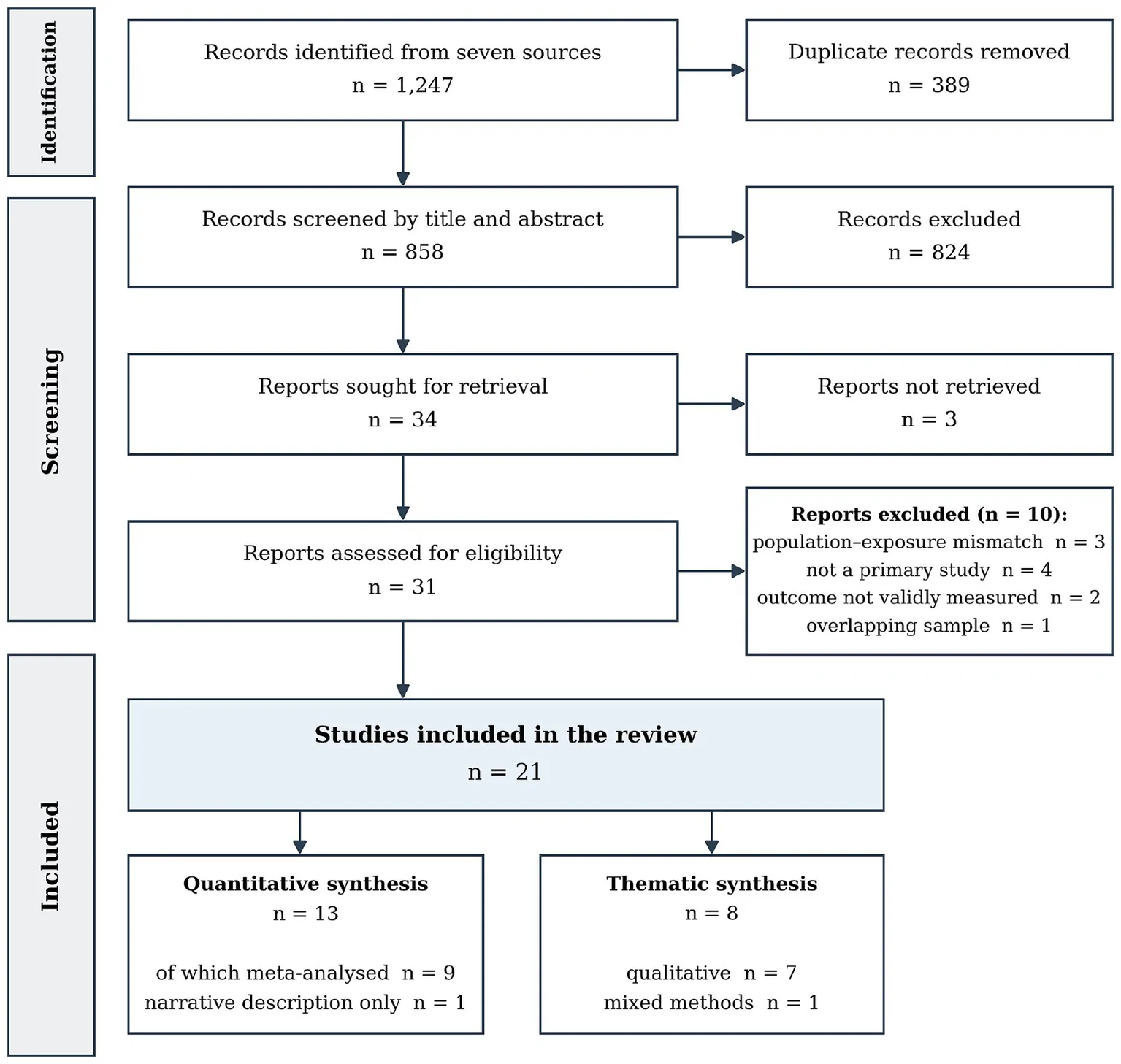
PRISMA 2020 flow diagram of study identification, screening, and inclusion. It shows the passage of records from database yield through de-duplication, title and abstract screening, full-text assessment and final inclusion, with counts and exclusion reasons at each stage.

### Characteristics of included studies

Bibliographic and design characteristics of the 13 included quantitative studies are presented in Table 1. Samples spanned the responder spectrum: firefighters engaged in urban search and rescue, National Medical Rescue Team personnel, hospital-based health professionals resident in the affected provinces, health workers deployed into the zone from unaffected provinces, hospital staff outside the zone treating evacuated casualties, child and adolescent psychiatrists, psychosocial support teams, social workers, and unaffiliated volunteers. All used non-probability sampling, most recruited online, and none reported a response rate against a defined sampling frame. Where reported, sex composition varied from 5.8 to 80.5% female (Table 1), reflecting the occupational structure of the respective professions. Intervals between the earthquakes and data collection ranged from 2 weeks to 22 months.

**Table 1:** Characteristics of included quantitative studies.

Study	Population	Provinces/setting	*N*	% female	Design	Months post	Deployment
Türk and Kaya (34)	Social workers, psychosocial support	Kahramanmaraş and Hatay zone	249	56.6	Cross-sectional	<6	Deployed
Bakirci, Sar and Cetin (6)	Firefighters, urban search, and rescue	Deployed to Pazarcık and Elbistan	223	5.8	Cross-sectional	6	Deployed
Topkara et al. (18)	Health professionals	Hatay	475	n.r.	Cross-sectional	3–6	Resident
Akdağ et al. (58)	Child and adolescent psychiatrists	Eleven declared provinces	41	80.5	Cross-sectional	17–18	Resident
Hugelius, Murphy and Blomberg (4)	Local and international responders	Türkiye and Syria	525	31.4	Cross-sectional	<6	Mixed
Sevim and Öksüz Poplata (37)	Unaffiliated and NGO volunteers	Deployed from İstanbul	101	58.4	Cross-sectional	2–3	Deployed
Etçioğlu et al. (27)	UMKE medical rescue personnel	Sakarya, deployed to zone	32 → 29	21.9ᵇ	Cohort, 4 waves	0.5–6	Deployed
Sisman, Cerit and Polat (33)	Psychosocial support teams	Deployed to affected zone	117	76.9	Cross-sectional	n.r.	Deployed
Koca, Deniz and İnceoğlu (38)	Hospital health personnel	Eleven declared provinces	315	58.4	Cross-sectional	2.5–3	Resident
Onat et al. (53)	Health workers treating evacuated casualties	Ankara (outside the zone)	159 of 243	74.1ᵃ	Cross-sectional	2–4	Distant
Taş et al. (39)	Health workers, affected and deployed	Five affected provinces	133 (71/62)	54.9	Cross-sectional	6	Both, contrasted
Kaya and Kara (32)	Psychosocial support professionals	Deployed to affected zone	341	70.4	Cross-sectional	6–10	Deployed
Derya et al. (40)	Physicians and nurses	Malatya	183	49.7	Cross-sectional	21–22	Resident

Instruments by study. Türk and Kaya: CF-SS, BRS. Bakirci et al.: PETLDS, DASS-21, PCL-5, BRS. Topkara et al.: STSS (Kâhil), CF-SS. Akdağ et al.: STSS (Yıldırım), CBI, PTSD-SS. Hugelius et al.: investigator-designed items. Sevim and Öksüz Poplata: STSS (Kâhil, 13-item modification), RSA. Etçioğlu et al.: DSM-5 ASSS, PCL-5, BAI, BDI. Sisman et al.: PCL-5 administered as a measure of secondary traumatic stress, CD-RISC. Koca et al.: PHS, CRI, PETSSS. Onat et al.: STSS (Yıldırım), BSI, WCSS. Taş et al.: PSS-SR, STSS (Yıldırım). Kaya and Kara: STSS (Kâhil). Derya et al.: PETLDS, CF-SS. Onat et al. contributed the subgroup of 159 participants who delivered care to earthquake casualties, and only these 159 enter the sample total of 2,894, Table 2, Figure 2 and every pooled estimate. The 84 who did not treat casualties do not meet the eligibility criteria and are recorded as an excluded comparison group in Supplementary File S5. They appear only in the Onat et al. rows of Tables 3, 4, because the source reported those contrasts and correlations for its full sample of 243 alone; these values are labelled as full-sample values, are presented descriptively and enter no pooled estimate. Hugelius et al. assessed outcomes with investigator-designed items without established psychometric properties and was retained for narrative description only. Sisman et al. administered the PCL-5 with explicit instruction to respond in relation to work with disaster survivors, so it indexes secondary traumatic stress rather than primary PTSD and contributes no prevalence data. % female = percentage of female participants in the analyzed sample. ᵃReported by the source for its full sample of 243 only; not available for the 159 eligible participants. 𝑺s reported for the enrolled sample of 32; the counts given alongside it (25 men, 5 women) do not reconcile with this total. n.r. = not reported. STSS = Secondary Traumatic Stress Scale (11); CF-SS = Compassion Fatigue Short Scale (14, 61); PETLDS = Post-earthquake Trauma Level Determination Scale (62); DASS-21 = Depression Anxiety Stress Scales-21 (63, 64); PCL-5 = PTSD Checklist for DSM-5 (65, 56); BRS = Brief Resilience Scale (66, 67); CBI = Copenhagen Burnout Inventory (15); PTSD-SS = PTSD Short Scale (57); RSA = Resilience Scale for Adults (68, 69); ASSS = Acute Stress Symptom Severity Scale (70); BAI = Beck Anxiety Inventory (71, 72); BDI = Beck Depression Inventory (73, 74); CD-RISC = Connor–Davidson Resilience Scale (75); PHS = Psychological Hardiness Scale (76); CRI = Coping Responses Inventory (77, 78); PETSSS = Post-Earthquake Traumatic Stress Screening Scale (79); PSS-SR = PTSD Symptom Scale Self-Report (80, 81); BSI = Brief Symptom Inventory (82, 83); WCSS = Ways of Coping with Stress Scale (84).

### Risk of bias

All 21 included studies were appraised and rated at moderate or low-to-moderate risk of bias overall; domain-level assessments are reported in Supplementary File S4. Common weaknesses were consistent across the set: every study used convenience, haphazard, snowball or self-selected sampling, none identified and addressed confounding, and only Etçioğlu et al. (27) employed a longitudinal design permitting inference about temporal ordering.

The verification procedure proved consequential and identified problems in 7 of the 13 quantitative studies. In Bakirci et al. (6), Post-earthquake Trauma Level Determination Scale subgroup medians are reported inverted relative to the instrument cut-off; the row of the second table labelled as DASS-21 total contains values identical to the PETLDS subgroup values and implies a score range contradicting the paper’s own; the resilience results paragraph reproduces the preceding PCL-5 paragraph verbatim; and the discussion reports depression and anxiety proportions that are not derivable from the paper’s own severity table. In Topkara et al. (18), the abstract reports the correlation between secondary traumatic stress and compassion fatigue as negative while the results table and text both give it as positive; the table value was extracted. In Onat et al. (53), the Ways of Coping subscales do not sum to the reported totals, and the optimistic and social-support rows carry identical values to two decimal places including the standard deviation; the Secondary Traumatic Stress Scale values are internally coherent and were used. In Türk and Kaya (34), sex-stratified secondary trauma means reweight to a value 0.29 above the reported overall mean, and the regression paragraph for parenthood duplicates the marital-status paragraph verbatim with a sign-flipped coefficient; the tables are coherent and were used. In Sevim and Öksüz Poplata (37), the marital status and parenthood percentages are transposed in the demographic table, and the reported resilience total cannot be reproduced from any subgroup breakdown, all of which converge on a different value; the secondary traumatic stress values reproduce exactly and were used. In Derya et al. (40), the departmental breakdowns for both principal scales overshoot the reported totals, while every other subgroup breakdown reconciles exactly; the totals were used. In Taş et al. (39), the Introduction dates the earthquakes to 2022 and one demographic percentage is misprinted; neither affects the extracted values.

Three studies report conflicting ethics approval details, giving two different dates or numbers within the same article: Bakirci et al. (6), Memiş Doğan and Sevinçli Bayram (54), and Kaya and Kara (32). This recurs often enough to be noted as a feature of the evidence base rather than an isolated defect. Two further values were judged non-extractable. Koca et al. (38) describe the Traumatic Stress Symptom Scale as 17 items scored 0–3 with a total range of 0–51, but report observed maxima of exactly 68 for both sexes, which is 17 items scored 1–4; the reported mean cannot be located on a defined metric and was excluded from synthesis pending clarification. Taş et al. (39) state that probable PTSD was present in 76.1% of affected health workers when a suggested cut-off is applied, but no cut-off value is specified and the Turkish validation they cite establishes none; this proportion was excluded from the prevalence meta-analysis.

### Secondary traumatic stress

Four studies measured the primary outcome with the 17-item Secondary Traumatic Stress Scale scored from 17 to 85 and reported a mean with a standard deviation, contributing 1,016 responders. A fifth, Taş et al. (39), reported medians with interquartile ranges for two exposure groups and was converted for the sensitivity and subgroup analyses. Individual and pooled estimates are presented in Figure 2, and all extracted outcome values across studies are reported in Table 2.

**Figure 2:**
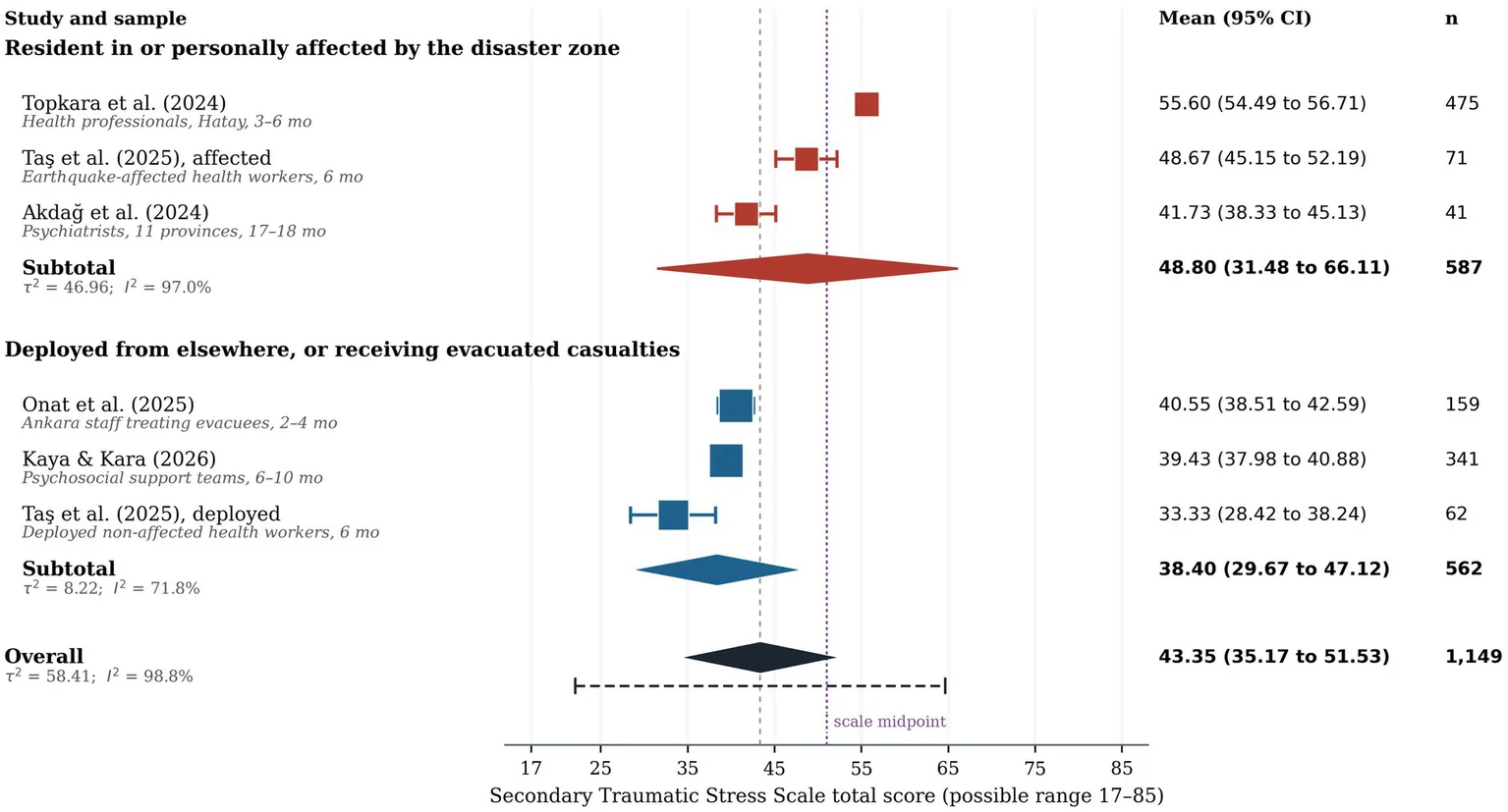
Secondary Traumatic Stress Scale total among responders to the 2023 Kahramanmaraş earthquakes, by personal exposure to the disaster zone. Squares are individual estimates with area proportional to study weight; horizontal lines are 95% confidence intervals. Diamonds are random-effects subgroup and overall estimates; the dashed line beneath the overall diamond is the 95% prediction interval. Random-effects models with restricted maximum likelihood estimation and Hartung–Knapp–Sidik–Jonkman adjustment. The vertical grey dashed line marks the overall pooled estimate and the purple dotted line the scale midpoint (51). Values for Taş et al. (39) were estimated from reported medians and interquartile ranges using the method of Wan et al. (43). The overall value for Kaya and Kara (32) was reconstructed by the review authors from five independent subgroup breakdowns, which converged to within 0.01 of one another.

**Table 2:** Psychological outcomes across included quantitative studies.

Outcome	Study	*N*	Instrument (range)	Mean ± SD	% above cut-off [95% CI]	α
Secondary traumatic stress	Topkara et al. (18)	475	STSS total (17–85)	55.60 ± 12.30	—	0.922
	Taş et al. (39), affected	71	STSS total (17–85)	48.67 ± 15.14ᵃ	—	n.r.
	Akdağ et al. (58)	41	STSS total (17–85)	41.73 ± 11.11	63.4 [48.1–76.4] moderate–severe	0.912
	Onat et al. (53)	159	STSS total (17–85)	40.55 ± 13.10	—	n.r.
	Kaya and Kara (32)	341	STSS total (17–85)	39.43 ± 13.70ᵇ	—	0.940
	Taş et al. (39), deployed	62	STSS total (17–85)	33.33 ± 19.73ᵃ	—	n.r.
	Sevim and Öksüz Poplata (37)	101	STSS, 13-item modificationᶜ	31.81 ± 11.87	—	0.905
	Sisman et al. (33)	117	PCL-5 as STS (0–80)	27.73 ± 15.26	—	n.r.
Compassion fatigue	Topkara et al. (18)	475	CF-SS total (13–130)	71.70 ± 28.30	—	0.939
	Derya et al. (40)	183	CF-SS total (13–130)	60.12 ± 25.95	—	0.920
	Türk and Kaya (34)	249	CF-SS total (13–130)	51.61 ± 24.18ᵈ	—	0.900
Probable PTSD	Bakirci et al. (6)	223	PCL-5 (0–80)	20.5 ± 18.3	16.1 [11.9–21.5] ≥ 41	n.r.
	Etçioğlu et al. (27)	32 → 29	PCL-5 (0–80)	n.r.	12.5 [5.0–28.1] ≥ 47 at 2 wk.; 3.4 at 6 mo	n.r.
	Akdağ et al. (58)	41	PTSD-SS (0–36)	10.02 ± 6.99	9.8 [3.9–22.5] ≥ 24	0.903
Post-traumatic stress	Taş et al. (39), affected	71	PSS-SR (0–51)	Md 27.0 (16.0–34.0)	—	n.r.
	Taş et al. (39), deployed	62	PSS-SR (0–51)	Md 14.0 (5.0–29.0)	—	n.r.
Earthquake trauma	Derya et al. (40)	183	PETLDS (20–100)	50.75 ± 14.31	—	0.890
	Bakirci et al. (6)	223	PETLDS (20–100)	37.5 ± 11.6	12.6 [8.8–17.5] ≥ 52.4	n.r.
Depression	Bakirci et al. (6)	223	DASS-21 depression	—	43.5 mild+; 21.1 moderate+	n.r.
	Etçioğlu et al. (27)	29	BDI	n.r.	27.6 [14.7–45.7] ≥ 17 at 6 mo	n.r.
Anxiety	Bakirci et al. (6)	223	DASS-21 anxiety	—	65.5 mild+; 48.9 moderate+	n.r.
	Etçioğlu et al. (27)	29	BAI	n.r.	13.8 at 6 mo	n.r.
Stress/acute stress	Bakirci et al. (6)	223	DASS-21 stress	—	83.0 mild+; 32.7 moderate+	n.r.
	Etçioğlu et al. (27)	32 → 29	DSM-5 ASSS (0–28)	n.r.	21.9 at 2 wk.; 17.2 [7.6–34.5] at 6 mo	n.r.
Burnout	Derya et al. (40)	183	CF-SS burnout (8–80)	38.85 ± 16.51	—	0.860
	Türk and Kaya (34)	249	CF-SS burnout (8–80)	32.24 ± 15.28ᵈ	—	0.860
	Akdağ et al. (58)	41	CBI work-related (0–100)	49.27 ± 18.63	48.8 [34.3–63.5] moderate–high	0.916
Resilience	Sisman et al. (33)	117	CD-RISC	63.24 ± 12.53	—	n.r.
	Bakirci et al. (6)	223	BRS	21.1 ± 4.0	—	n.r.
	Sevim and Öksüz Poplata (37)	101	RSA (33–165)	130.67 ± 16.03ᵇ	—	0.930

𝐾stimated from reported medians and interquartile ranges using Wan et al. (43). ᵇReconstructed by the review authors from subgroup breakdowns; the source reports no overall value for Kaya and Kara, and the reported overall value in Sevim and Öksüz Poplata is not reproducible from its own subgroups. ᶜFour items were removed after the authors’ own factor analysis, so this total is not on the 17–85 metric and did not enter the meta-analysis. ᵈRescaled by the review authors from reported item means by multiplication by the number of items. n.r. = not reported. Proportions carry Wilson score confidence intervals computed by the review authors. α = Cronbach’s alpha as reported in the source study. Instrument references are given in the legend of Table 1. Values from Koca et al. (38) are omitted because the response metric of the Traumatic Stress Symptom Scale could not be established from the source; see Supplementary File S4.

The pooled Secondary Traumatic Stress Scale total was 44.37 (95% CI 32.25–56.50), with τ^2^ = 57.54, *Q*(3) = 379.2, *p* < 0.001, *I*^2^ = 99.2% and a 95% prediction interval of 17.36–71.39. Adding Taş et al. as its two exposure groups gave a pooled estimate of 43.35 (35.17–51.53) across 6 groups. A fixed-effect model returned 47.89. This study reports this pooled mean for completeness and does not interpret it as an estimate of secondary traumatic stress in this workforce. The confidence interval spans 32 points on a 68-point scale and *I*^2^ approaches unity; a single summary mean is not a meaningful quantity here. The informative result is what accounts for that dispersion.

As Figure 2 shows, splitting the six exposure groups by whether personnel were resident in or personally affected by the disaster zone produced a separation in the expected direction. Personnel who were themselves in the zone scored 48.80 (95% CI 31.48–66.11), and personnel deployed from elsewhere or treating evacuated casualties scored 38.40 (29.67–47.12), a difference of 10.40 points with an approximate 95% confidence interval of 1.57–19.23. This interval is wide, and the estimate should be read with corresponding caution. Its lower bound, approximately 1.6 points on the 68-point range of the instrument and about one-eighth of a typical within-study standard deviation of 13 points, would be of doubtful practical significance, whereas its upper bound would correspond to roughly one and a half standard deviations. The direction of the contrast is therefore better supported than its magnitude, which cannot be estimated precisely from six groups drawn from five studies. The heterogeneity statistics are as informative as the point estimates. Between-study variance fell from τ^2^ = 46.96 in the affected subgroup to τ^2^ = 8.22 in the deployed subgroup, and *I*^2^ from 97.0 to 71.8%. Once personal exposure is conditioned upon, the deployed samples behave as a reasonably coherent population despite spanning social workers, psychosocial support teams and hospital clinicians surveyed between 2 and 10 months after the event.

The instrument-adaptation moderator can be reported as null. Studies using the Kâhil (12) adaptation returned means of 39.43 and 55.60; studies using the Yıldırım et al. (13) adaptation returned 40.55, 41.73, and 42.00. The two adaptations do not separate, and translation differences therefore do not account for the spread. Interval since the event could not be modelled formally at this number of studies, but the two highest values come from the samples closest to the epicenter and the lowest from a deployed group, while the resident sample surveyed latest, at 17–18 months, sits with the deployed studies rather than with the resident ones.

### Compassion fatigue

Three studies measured compassion fatigue with the 13-item Compassion Fatigue Short Scale on the 13–130 metric, contributing 907 responders. The pooled total was 61.17 (95% CI 36.05–86.29), with τ^2^ = 100.27, *Q*(2) = 102.2, *p* < 0.001 and *I*^2^ = 98.0%. The ordering again follows proximity: health professionals resident in Hatay scored highest at 71.70, physicians and nurses in Malatya intermediate at 60.12, and social workers deployed into the zone lowest at 51.61. The pattern is consistent with the primary outcome, but at three studies it should be read as convergent rather than as independent confirmation. The secondary-trauma subscale of the same instrument was reported by two studies only, at 21.26 (SD 10.74) among physicians and nurses and 17.80 (SD 8.35) among social workers, and remained below the threshold for pooling.

### Pooled prevalence of probable PTSD

Three studies reported the proportion of responders exceeding a validated threshold for probable PTSD, using three different instrument–threshold combinations: the PCL-5 at 41, recommended for first responders (55); the PCL-5 at 47, the threshold recommended in the Turkish validation (56); and the 9-item PTSD Short Scale at 24 (57). Across 296 responders, 44 exceeded threshold. Individual and pooled estimates are presented in Figure 3. The pooled prevalence was 14.5% (95% CI 8.5–21.5), with τ^2^ = 0.000, *Q*(2) = 1.02, *p* = 0.599 and *I*^2^ = 0.0%. Because between-study variance was estimated at exactly zero, the 95% prediction interval coincides with the confidence interval and conveys no additional information; that coincidence is itself a caution, since it reflects an estimate of τ^2^ that three studies cannot support rather than genuine consistency across future samples.

**Figure 3:**
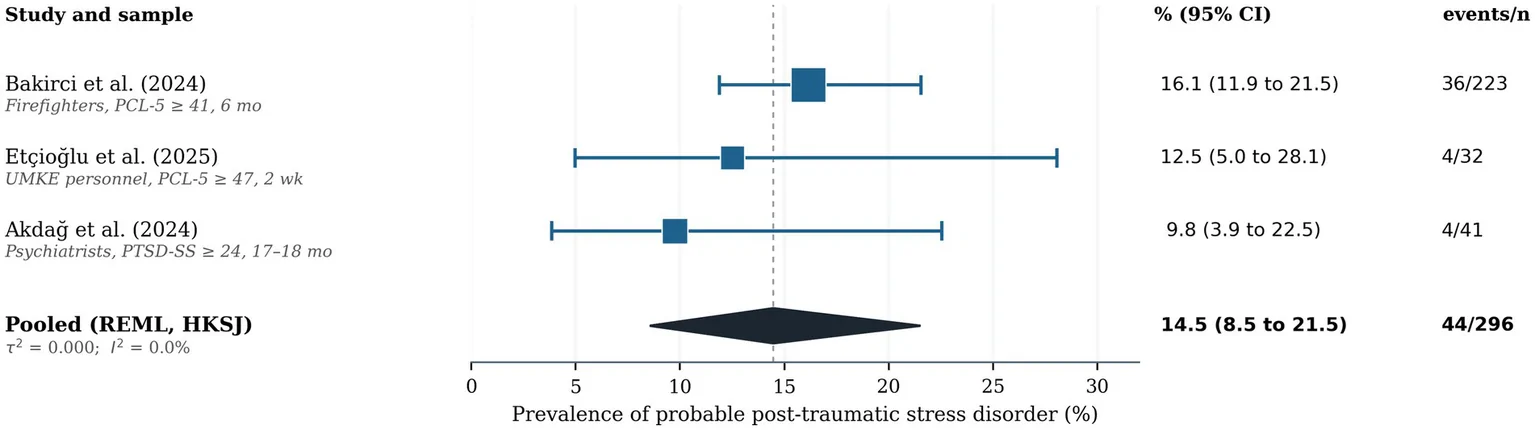
Pooled prevalence of probable post-traumatic stress disorder among responders to the 2023 Kahramanmaraş earthquakes. Squares are individual study estimates with area proportional to study weight; horizontal lines are Wilson score confidence intervals. The diamond is the pooled random-effects estimate and the vertical dashed line marks the pooled value; because τ^2^ was estimated at zero, the 95% prediction interval coincides with the confidence interval and is not drawn separately. Random-effects model with restricted maximum likelihood estimation, Hartung–Knapp–Sidik–Jonkman adjustment, and Freeman–Tukey double arcsine transformation.

As Figure 3 shows, heterogeneity was undetectable on this outcome, and the estimate was robust to the exclusion of any single study, with leave-one-out values of 10.9, 14.9, and 15.3%; a fixed-effect model returned an identical 14.5%. Two cautions apply and both are material. *I*^2^ is estimated imprecisely at three studies, and a value of zero should be read as absence of evidence of heterogeneity rather than as evidence of homogeneity (51). The prediction interval is uninformative for the reason just given, so no statement about a future sample can be supported (52). This estimate rests on the narrowest evidence base of the three pooled outcomes and, unlike the primary outcome, draws only on deployed and mixed samples; it should be regarded as provisional. Small-study effects could not be assessed.

### Personal earthquake exposure as a modifier of outcome

The subgroup contrast reported above is a between-study comparison. It is reproduced within studies, and with a consistent ordering by severity of loss. Within the largest contributing sample, personal earthquake exposure predicted secondary traumatic stress in graded fashion, with effect sizes computed by the review authors from the subgroup means reported by Topkara et al. (18) and presented in Table 3. Responders physically injured in the earthquake scored almost a full standard deviation higher than those who were not; responders whose homes were destroyed approximately two-thirds of a standard deviation higher; and bereaved responders approximately two-fifths of a standard deviation higher. The same ordering was reproduced for compassion fatigue for physical injury and home destruction, although the bereavement contrast did not reach significance on that outcome.

**Table 3:** Personal and family earthquake exposure and psychological outcome within two samples.

Outcome	Exposure	*n* (exposed vs not)	Exposed, M ± SD	Not exposed, M ± SD	Hedges’ *g*	95% CI	*p*
Topkara et al. (18), *N* = 475 — responder’s own exposure							
Secondary traumatic stress	Physical injury	52 vs. 423	66.0 ± 13.7	54.3 ± 11.5	0.99	0.70–1.29	<0.001***
	Home destroyed	60 vs. 415	63.0 ± 12.7	54.6 ± 11.9	0.70	0.42–0.97	<0.001***
	Bereavement	179 vs. 296	58.7 ± 12.1	53.7 ± 12.1	0.41	0.23–0.60	<0.001***
Compassion fatigue	Physical injury	52 vs. 423	91.3 ± 31.1	69.3 ± 27.0	0.80	0.51–1.09	<0.001***
	Home destroyed	60 vs. 415	83.6 ± 31.2	70.0 ± 27.4	0.49	0.21–0.76	<0.001***
	Bereavement	179 vs. 296	73.8 ± 30.3	70.4 ± 26.9	—	Not significant	0.198
Onat et al. (53), full source sample *N* = 243 (159 eligible responders and 84 ineligible comparison staff)—exposure of the responder’s relatives							
Secondary traumatic stress	Relative died	13 vs. 230	46.62 ± 15.35	38.50 ± 12.93	0.62	0.06–1.18	0.067
	Relative injured	19 vs. 224	43.84 ± 14.10	38.52 ± 13.03	0.40	−0.07 to 0.87	0.091
	Relative financial or shelter loss	81 vs. 162	40.33 ± 12.54	38.24 ± 13.44	0.16	−0.11 to 0.43	0.191
	Treated earthquake casualties	159 vs. 84	40.55 ± 13.10	35.89 ± 12.81	0.36	0.09–0.62	0.008**

Effect sizes computed by the review authors from subgroup means reported in the source articles; p values as reported in the source. Hedges’ g incorporates the small-sample correction (45). The two panels concern different constructs and are not pooled: Topkara et al. record the responder’s own injury, bereavement, and property loss, whereas Onat et al. record the same events occurring to the responder’s relatives. The Onat et al. panel is reported for the full source sample because the source did not report these contrasts for its 159 eligible participants separately; it therefore includes the 84 staff who did not treat casualties and are otherwise excluded from the review (see legend of Table 1 and Supplementary File S5), and its final row contrasts the eligible subgroup with that ineligible group. These values are descriptive and enter no pooled estimate. **p < 0.01; ***p < 0.001.

Table 3 shows the same ordering by severity of loss in both panels, and it holds in Onat et al. on its full source sample even though the losses there were sustained by relatives rather than by the responder. Two further replications support the pattern without permitting effect size computation. Akdağ et al. (58) found that psychiatrists whose homes were damaged and those bereaved in the earthquake scored significantly higher on both secondary traumatic stress and post-traumatic stress symptoms, but reported no subgroup means. Derya et al. (40) found higher post-earthquake trauma levels among clinicians who lost a relative, relocated because of home damage, could not contact a loved one, or experienced nutritional problems on duty, and higher secondary trauma among those who could not contact a loved one. Kaya and Kara (32) found higher secondary traumatic stress among professionals with a personal trauma history than among those without. Neither the primary studies nor this synthesis adjusts for confounding, and in every case the finding emerges from unadjusted subgroup comparison. The convergence across six independent samples of markedly different professional composition, surveyed between 2 and 22 months apart, is nonetheless notable. It is worth adding that in the largest sample, prior earthquake experience and prior service in a disaster zone were both unrelated to secondary traumatic stress while personal loss in this earthquake was strongly related, a contrast suggesting that personal victimization rather than cumulative occupational exposure carries the risk.

### Organizational conditions of deployment

Two studies converge on a finding with direct operational implications. Kaya and Kara (32) found that psychosocial support professionals assigned to the disaster zone by their institutions scored substantially higher on secondary traumatic stress than those who volunteered, at 43.04 against 36.73 (*t* = 4.170, *p* < 0.001, *d* = 0.46), the largest single contrast in that study and larger than the effect of sex, profession, institution type, or professional experience, none of which reached significance. Türk and Kaya (34) found the same for occupational burnout among social workers, at 4.56 against 3.60 on the item metric, and further reported that the association between burnout and resilience was markedly stronger among assigned personnel (*r* = −0.709) than among volunteers (*r* = −0.342). Kaya and Kara also found a dose relationship for time in the field: professionals who spent 8–14 days in the zone scored lowest at 36.01, against 42.90 for those deployed 22–28 days and 41.54 for those deployed 29 days or more. Both variables are organizational rather than dispositional, and both are modifiable.

### Symptom severity, trajectory, and other outcomes

Symptom severity and categorical outcomes for all remaining domains are reported in Table 2. Among firefighters at 6 months the mean PCL-5 total was 20.5 (SD 18.3) and the mean PETLDS 37.5 (SD 11.6), with 12.6% (8.8–17.5) exceeding the earthquake trauma threshold (6); among physicians and nurses in Malatya at 21–22 months the PETLDS mean was considerably higher at 50.75 (SD 14.31) (40), the second study to use that instrument. The only prospective evidence comes from Etçioğlu et al. (27), who followed 32 National Medical Rescue Team personnel at 2 weeks and at 1, 3, and 6 months. Above-threshold PCL-5 scores fell from 12.5% (5.0–28.1) to 3.4%, and above-threshold acute stress from 21.9 to 17.2% (7.6–34.5), with attrition from 32 to 29 participants. Omnibus tests across timepoints were non-significant for every instrument, which the sample size renders unsurprising rather than informative. Depression, by contrast, did not decline monotonically and remained above threshold in 27.6% (14.7–45.7) of personnel at 6 months, while anxiety stood at 13.8%. Recomputation from the firefighter severity table gives 43.5% at or above mild and 21.1% at or above moderate for depression, 65.5 and 48.9% for anxiety, and 83.0 and 32.7% for stress. Resilience was measured on four non-comparable instruments and could not be pooled; notably, Sevim and Öksüz Poplata (37) found no significant association between resilience and secondary traumatic stress among volunteers, whereas Koca et al. (38) and Türk and Kaya (34) both reported significant negative associations in personnel with a formal institutional role. No study in the extracted set reported compassion satisfaction or post-traumatic growth. Hugelius et al. (4) surveyed 525 local and international responders but with investigator-designed items, so their findings are not comparable with the above; they nonetheless reported that local responders perceived significantly more health problems than international responders, which is consistent in direction with the exposure gradient reported here.

### Associations among outcomes

Reported correlations are presented in Table 4 and were consistent in direction across every fully extracted study. Earthquake-specific trauma level correlated strongly with general distress and moderately with post-traumatic stress, and negatively with resilience. Post-traumatic stress correlated very strongly with secondary traumatic stress but only weakly with burnout. Secondary traumatic stress correlated strongly with compassion fatigue, and both correlated negatively with resilience.

**Table 4:** Reported correlations among psychological constructs.

Study	*N*	Constructs	*r*
Türk and Kaya (34)	249	Secondary trauma—burnout	0.703**
		Resilience—compassion fatigue	−0.604**
		Resilience—burnout	−0.579**
		Resilience—secondary trauma	−0.489**
Bakirci et al. (6)	223	PETLDS—DASS-21	0.704**
		PETLDS—PCL-5	0.572**
		PETLDS—BRS	−0.540**
		DASS-21—PCL-5	0.539**
Akdağ et al. (58)	41	PTSD-SS—STSS	0.802**
		STSS—CBI	0.432**
		PTSD-SS—CBI	0.334*
Topkara et al. (18)	475	STSS—CF-SS	0.739**
Onat et al. (53)	243ᵃ	STSS—anxiety	0.670**
		STSS—depression	0.650**
		STSS—ineffective coping	0.480**
		STSS—effective coping	−0.380**
Taş et al. (39)	133	STSS—PSS-SR	0.878**
Koca et al. (38)	315	Resilience—coping with stress	0.520**
		Resilience—traumatic stress	−0.281**
Derya et al. (40)	183	PETLDS—CF-SS	0.654**
		PETLDS—burnout	0.650**
		PETLDS—secondary trauma	0.579**

Instrument abbreviations are given in the legend of Table 1. 𝐿ull source sample, including 84 staff who did not treat casualties and are otherwise excluded from the review; the source did not report these correlations separately for its 159 eligible participants. *p < 0.05; **p < 0.01.

Table 4 shows a consistent gap between the association of post-traumatic stress with secondary traumatic stress on the one hand, and with burnout on the other. Secondary traumatic stress tracks traumatic exposure closely and shares symptom structure with PTSD—the correlation with a validated PTSD self-report measure reaches 0.878 in Taş et al. (39)—whereas burnout appears more responsive to organizational conditions than to traumatic content. Onat et al. (53) add a third element: secondary traumatic stress correlates as strongly with anxiety and depression as it does with coping style, suggesting that the affective burden and the trauma burden are not separable in this workforce.

### Outcomes not meeting the threshold for pooling

Beyond secondary traumatic stress, compassion fatigue and probable PTSD prevalence, no outcome in the extracted set was reported by three or more studies on a comparable metric. Depression, anxiety, and acute stress each reached two studies; earthquake-specific trauma level reached two; burnout reached three but across two incompatible instruments; resilience reached five across four incompatible instruments. These were synthesized narratively following Campbell et al. (42). Where pooling at two studies was attempted for documentation, the resulting confidence intervals fell outside the possible score range of the instrument in both directions, which is the correct behavior of the estimator rather than an artifact, since between-study variance cannot be estimated from two studies and the Hartung–Knapp adjustment propagates that ignorance rather than concealing it (47, 52).

### Qualitative synthesis

Eight qualitative and mixed-method reports contributed to the thematic synthesis; their characteristics are summarized in Table 5. They address search-and-rescue personnel and first-response teams, volunteer nurses, hospital nurses working in the affected provinces, disaster nursing more broadly, Turkish Red Crescent operations, and a mixed responder cohort interviewed 12 days after the earthquakes.

**Table 5:** Characteristics of qualitative and mixed-method reports contributing to thematic synthesis.

Study	Population	Provinces	*N*	Design	Focus
Koçak et al. (59)	Turkish Red Crescent personnel	National, all affected provinces	683	Mixed methods, retrospective	Organizational disaster response
Altunbek, Ildırım, and Atalar (85)	Disaster response personnel (of 105 interviewed)	Seven affected provinces, 14 sites	39	Unstructured interviews, thematic	Cognitive, emotional, and social experience at 12 days
Memiş Doğan, and Sevinçli Bayram (54)	First-response teams (UMKE, AFAD, AKUT)	Eleven affected provinces	12	Qualitative, thematic	Motivation, field experience, daily life afterwards
Çakaloğlu et al. (19)	Search-and-rescue volunteers	Not specified in source	21	Phenomenological	Psychological well-being and resilience
Çorbacı, Tansel, and Alkan (16)	Volunteers in search-and-rescue activities	TRNC-based, deployed to Türkiye	8	Phenomenological	Secondary trauma experiences
Celebi Cakiroglu et al. (17)	Volunteer nurses in the earthquake zone	Southeastern affected region	16	Interpretative phenomenological	Traumatic life experiences and their effects
Parlak et al. (60)	Local and volunteer nurses, first days	Affected provinces and volunteers from elsewhere	16	Phenomenological, inductive	Working conditions after the earthquakes
Mert and Koksal (86)	Disaster nurses, volunteers in the zone	Affected provinces	15	Qualitative, descriptive	Motivations, challenges, and lessons

Altunbek et al. interviewed 105 participants of whom 39 were disaster response personnel; only that subgroup contributes here. Koçak et al. administered structured surveys combining quantitative metrics with free-text qualitative feedback to 683 Turkish Red Crescent personnel; only the qualitative component contributes to this synthesis. Together these eight reports contribute data on 810 responders.

Eight themes were identified, spanning the nature of the exposure, the organizational conditions under which responders worked, the interpersonal resources available to them, and the modifiability of the resulting strain. They are presented in Table 6.

**Table 6:** Thematic synthesis of qualitative evidence.

Theme	Description	Sources
Dual helper–victim role	Responders resident in the affected provinces sustained bereavement and property loss while on duty, producing a conflict between the affect of helping and that of personal victimization.	Çakaloğlu et al. (19), Çorbacı et al. (16), Celebi Cakiroglu et al. (17)
Exposure to human remains and identification with victims	Contact with the deceased, confined-space extrication, and imagining oneself or one’s family in the victim’s place are described as the most distressing features of the work.	Çorbacı et al. (16)
Guilt at insufficiency	An unprompted and near-universal account among first responders: guilt at leaving the zone, at being unable to reach everyone, and at eating or resting while survivors could not. Described as distinct from both exhaustion and trauma symptoms.	Memiş Doğan and Sevinçli Bayram (54)
Organizational sources of avoidable strain	Role uncertainty, inadequate deployment planning, equipment and machinery shortages, extended shifts without rest, and failures of intra- and inter-organizational communication recur across accounts. Untrained volunteers on the rubble are repeatedly described as a source of secondary danger.	Memiş Doğan and Sevinçli Bayram (54), Parlak et al. (60), Koçak et al. (59), Yikilmaz et al. (9)
Rest, support, and leadership as protective	Turkish Red Crescent personnel identified rest and organizational support as essential and their absence as a primary stressor; servant leadership was associated with lower emotional exhaustion, mediated by workplace buoyancy.	Koçak et al. (59), Yikilmaz et al. (9)
Team cohesion and peer support	Group cohesion, supportive team interaction, and peer debriefing are described as the principal buffers available in the field, often substituting for absent formal provision.	Celebi Cakiroglu et al. (17), Mert and Koksal (86)
Burnout and exhaustion during the acute phase	Physical exhaustion, emotional depletion, absence of rest and recovery, and the conflict between duty and personal well-being were already being described by response personnel 12 days after the earthquakes.	Altunbek et al. (85)
Amenability to early intervention	Psychological first aid delivered to volunteers reduced involuntary exposure symptoms and increased resilience, indicating that the strain described is modifiable.	Bekircan et al. (35)

Every theme is attributed only to primary reports in which it could be located directly; no theme is attributed through a secondary source.

Three features of this material bear on the quantitative findings. The dual helper–victim role recurs across the qualitative accounts as a distinct source of strain and is described in terms of affective conflict rather than additive exposure, which corresponds directly to the subgroup separation in Figure 2. Guilt at insufficiency emerges unprompted and is not captured by any instrument used anywhere in the quantitative literature on this event, which suggests that the available measures may understate the burden they are intended to quantify. And the determinants that responders themselves identify as most consequential are predominantly organizational and interpersonal rather than intrapsychic, which corresponds to the correlational pattern in Table 4 and to the volunteer–assignee contrast reported above.

## Discussion

The central finding of this review is not a level but a boundary. Personnel who responded to the 2023 Kahramanmaraş earthquakes from inside the disaster zone score roughly 10 points higher on the Secondary Traumatic Stress Scale than personnel who came in from outside or treated evacuated casualties elsewhere, a point estimate of about three-quarters of a within-study standard deviation. That estimate is imprecise: its 95% confidence interval, 1.57–19.23 points, extends from a difference too small to matter in practice to one of about one and a half standard deviations. The pooled mean for the workforce as a whole, 44.37, is not a quantity this study would ask anyone to use: its confidence interval spans half the instrument and *I*^2^ approaches unity. However, that dispersion is not noise. Once personal exposure is conditioned upon, between-study variance in the deployed subgroup falls by more than four-fifths and *I*^2^ from 97 to 72%, and samples as different as social workers, psychosocial support teams, and hospital clinicians surveyed months apart begin to look like one population. The heterogeneity that has made this literature difficult to synthesize appears, to a substantial degree, to be exposure heterogeneity.

That conclusion is supported from several directions at once. It appears between studies in Figure 2. It appears within a single study in Taş et al. (39), who by design compared affected resident health workers with health workers deployed from unaffected provinces using the same instruments and found a 20-point separation in median score. It appears within samples as a dose relationship in Topkara et al. (18), where effect sizes for secondary traumatic stress order themselves by severity of personal loss from physical injury through home destruction to bereavement, and it is reproduced for compassion fatigue on the first two of those contrasts. It appears in attenuated form in Onat et al. (53), where the same ordering holds even when the losses were sustained by the responder’s relatives rather than by the responder, although those contrasts were reported only for the full source sample, which includes staff who did not treat casualties. And it is corroborated without effect sizes in Akdağ et al. (58), Derya et al. (40) and Kaya and Kara (32). Six independent samples, spanning firefighting, medicine, psychiatry, social work and psychosocial support, and surveyed between 2 and 22 months after the event, point the same way. What this convergence establishes with reasonable confidence is the direction of the effect. Its between-study magnitude remains imprecise, and the within-study contrasts in Topkara et al. (18), whose confidence intervals are considerably narrower, give the more secure indication of size.

This bears directly on how responder mental health is conceptualized and measured. Secondary traumatic stress is defined by indirect exposure (10) and its instruments are constructed on that premise (11). Yet in a disaster of this geographic scale a large share of the responding workforce was drawn from the affected provinces, and for those personnel the boundary between primary and secondary exposure is not clean. The correlation of 0.878 between the Secondary Traumatic Stress Scale and a validated PTSD self-report measure in Taş et al. (39) makes the point starkly: in a sample where many respondents were themselves victims, the two instruments are close to measuring the same thing. The international literature has approached this obliquely. Grossi et al. (8) report that being personally affected functions as a risk factor in some samples, that participation in relief work may be beneficial for aid workers who are themselves survivors, and that local rescuers report lower well-being than externally supporting rescuers; Hugelius et al. (4) found the same directional pattern in this event, with local responders perceiving significantly more health problems than international ones. The present synthesis offers a first, if imprecise, quantification of it. Three implications follow. Measurement should distinguish primary from secondary exposure explicitly rather than assuming the latter. Personal loss should be reported as a matter of course, as deployment duration currently is. And task allocation in the acute phase should take account of it, since assigning bereaved or injured personnel to the most exposure-intensive roles carries a foreseeable cost, even if its size cannot yet be estimated precisely.

The affective outcomes tell a related story, and the contrast with the unremarkable pooled PTSD prevalence makes them even more interesting. Depression exceeded threshold in more than a quarter of medical rescue personnel at 6 months and, unlike acute stress and post-traumatic stress in the same cohort, showed no clear decline across follow-up (27). Recomputed severity distributions among firefighters place anxiety at or above moderate in almost half the sample (6), well above the 20–30% range that study’s authors cite from comparable populations. In Onat et al. (53), secondary traumatic stress correlates as strongly with anxiety and depression as with anything else measured. Several explanations deserve consideration, and they are not mutually exclusive. The first is direct exposure. A substantial share of this workforce experienced the earthquakes as victims rather than only as witnesses, and depressive symptomatology is the expected sequela of loss rather than of threat. Bereavement, injury, and destruction of the home are grief exposures, and grief does not attenuate on the timescale over which intrusive and hyperarousal symptoms typically remit. The gradient reported in Table 3 supports this reading directly, since it is severity of personal loss rather than intensity of operational exposure that orders the effect sizes. The second is timing of measurement. Acute stress and post-traumatic stress were assessed from two weeks onward, when they are at their peak and their natural course is toward decline, whereas depression in disaster-affected populations characteristically emerges later and persists as the practical consequences of loss consolidate. The third is occupational stress that has nothing to do with traumatic content: extended shifts, absent rest provision, equipment shortage, role uncertainty, and communication failure recur across the qualitative accounts (54, 59, 60), and these are the conditions under which demoralization rather than post-traumatic stress develops. The fourth is the specific position of local health personnel, who served simultaneously as responders and as disaster victims while continuing to work in damaged facilities and to live with the consequences of the event outside their shifts. Koca et al. (38) found that psychological resilience exerted a significant negative effect on traumatic stress symptoms among 315 hospital staff across the 11 provinces, and Derya et al. (40) found that a third of clinicians were considering leaving their profession because of what they had experienced—an occupational consequence that symptom counts do not capture. What distinguishes this responder workforce may therefore not be trauma-related morbidity but the affective and occupational burden that accompanied it. This diverges from the emphasis of the international responder literature, which has concentrated heavily on post-traumatic stress; Grossi et al. (8) make a similar observation, and Ruhm et al. (2) reach it independently in noting that the burnout literature for this event documents burden extensively while developing almost no structured means of preventing it.

A related distinction concerns the targets of intervention, and the evidence for it has strengthened considerably. Secondary traumatic stress correlates very strongly with post-traumatic stress symptoms and strongly with compassion fatigue, while burnout correlates only weakly with post-traumatic stress. Two studies now show that burnout and secondary traumatic stress are both higher among personnel assigned to the zone by their institutions than among those who volunteered—Kaya and Kara (32) for secondary traumatic stress, with the largest effect in their study, and Türk and Kaya (34) for burnout, with the association between burnout and resilience nearly twice as strong among assignees as among volunteers. Kaya and Kara further report a dose relationship with time in the field, with the lowest scores at 8–14 days and higher scores beyond 3 weeks. These are organizational variables, not dispositional ones, and unlike sex or trauma history they are under institutional control. This is consistent with the conceptual separation of burnout as a response to chronic occupational demand (15) from trauma-linked symptomatology, and with the emphasis Grossi et al. (8) place on organizational and social determinants of helper resilience. Interventions designed around trauma processing are unlikely to address burnout, and improvements in organizational practice unlikely to address secondary traumatic stress; provision that treats responder distress as a single construct will under-serve one or the other. That the strain is modifiable is indicated by Bekircan et al. (35), who found that psychological first aid delivered to volunteers after these earthquakes reduced involuntary exposure symptoms and increased resilience.

The trajectory evidence, though resting on a single small cohort, carries a direct implication for practice. If acute stress and post-traumatic stress decline while depression persists, then post-deployment monitoring calibrated to trauma symptoms will detect the improving problem and overlook the stable one. Screening protocols that administer a PTSD checklist and nothing else would, on this evidence, miss the outcome most likely to remain. One further observation from that cohort deserves emphasis. Participants scoring above threshold were contacted, informed, and advised to consult a specialist, and none did so (27). This is consistent with the wider literature on help-seeking among health and emergency personnel, in which uptake is characteristically low and the reason most frequently given is a preference for resolving problems independently. It suggests that identification is not the binding constraint on responder mental health provision. How services are designed may matter more than the accuracy of screening: opt-out rather than opt-in, embedded rather than referred, delivered by peers rather than by clinicians.

The qualitative material adds one construct that the quantitative literature does not measure at all. Guilt at insufficiency emerged unprompted among first-response personnel—guilt at withdrawing from the zone, at being unable to reach everyone, at eating or resting while survivors could not (54). It is described as distinct from exhaustion and from intrusive symptoms, and it maps onto the moral injury literature rather than onto the trauma or burnout literatures. No instrument used in any of the 13 quantitative studies captures it. If it is as common as that account suggests, the available measures understate the burden they are intended to quantify, and the field has been counting the wrong things at least in part.

A methodological observation follows from the difficulty encountered in synthesis. Across 13 quantitative studies, secondary traumatic stress was assessed with two Turkish adaptations of the same scale plus one 13-item modification of it and one repurposed PTSD checklist; post-traumatic stress with four instruments and two thresholds on one of them; resilience with four non-comparable instruments; and burnout with two. Three studies reported item means rather than totals, one reported medians only, one reported no overall value at all, and one described a response metric that its own tables contradict. Three carried conflicting ethics approval details, and four contained duplicated or internally irreconcilable passages. This fragmentation is consequential here precisely because the volume of primary research on this event is otherwise sufficient to support quantitative synthesis: this study was able to pool three outcomes only after applying three separate reconstructive transformations, each of which introduces assumptions that a better-reported literature would not require. Convergence on the Secondary Traumatic Stress Scale and the PCL-5, with consistent thresholds, with totals reported alongside subscale scores, and with means and standard deviations reported alongside categorical results, would substantially increase the cumulative value of future work.

## Limitations

Several limitations qualify these findings. The pooled estimates rest on four studies for the primary outcome and three each for compassion fatigue and PTSD prevalence, which is at or near the minimum permitting estimation; prediction intervals are correspondingly wide. The exposure contrast is an unadjusted subgroup comparison across six groups drawn from five studies, not a meta-regression, and one of the six contributes values estimated from medians. Its 95% confidence interval, 1.57–19.23 points, is wide, and a difference at the lower bound would be of doubtful practical significance, so the direction of the contrast is better established than its size. The relatives’ exposure contrasts and correlations taken from Onat et al. (53) were reported by the source only for its full sample of 243, including 84 staff who did not treat casualties and are otherwise ineligible; they are presented descriptively and enter no pooled estimate. Three studies required reconstructive transformation before they could be pooled—rescaling from item means, estimation from medians, and reconstruction of a total from subgroup breakdowns—and each introduces assumptions recorded in Supplementary File S4. All contributing studies used non-probability sampling and recruited predominantly online, none adjusted for confounding, and all but one are cross-sectional. No study anywhere in this literature reported pre-deployment baselines, so no estimate of change from a personal baseline is available. The PTSD prevalence estimate draws only on deployed and mixed samples and is the least secure of the three. Unaffiliated volunteers remain under-represented, though less severely than in earlier syntheses. And this literature is growing quickly and is substantially Turkish-language, so any synthesis of it is provisional with respect to date of search.

## Conclusion

Whether a responder was also a victim was the most consistent modifier of psychological outcome after the 2023 Kahramanmaraş earthquakes, and it appears to account for much of the variation that has made this literature resistant to synthesis. The direction of this effect was consistent across between-study and within-study comparisons, but the between-study difference of 10.40 points was imprecisely estimated (95% CI 1.57–19.23), with a lower bound of doubtful practical significance, so its magnitude should be regarded as provisional. Approximately one in seven responders met criteria for probable PTSD, a prevalence consistent with international estimates for first responders, but the accompanying burden of depression, anxiety, burnout, and compassion fatigue was less consistent with those estimates and was concentrated in those with personal loss. Two further findings are directly actionable: personnel assigned to the disaster zone by their institutions carry more secondary traumatic stress and more burnout than those who volunteer, and deployments beyond three weeks carry more than deployments of 8–14 days. Three priorities for future research follow. Prospective designs with pre-deployment baselines are entirely absent from this literature and would resolve the central ambiguity about whether observed distress represents change from a personal baseline or a stable occupational characteristic. Personal exposure should be measured and reported routinely, with sufficient granularity to distinguish bereavement, injury, and property loss, since these carry different magnitudes. And guilt at insufficiency, which responders describe unprompted and no current instrument captures, warrants dedicated measurement. For practice, disaster mental health provision for this workforce should be organized around personal exposure rather than professional role alone; task allocation and deployment length should be treated as modifiable determinants of outcome; post-deployment monitoring should extend beyond trauma symptoms to depression; and services should be designed on the assumption that identification will not by itself produce uptake.

## Data Availability

The extraction dataset and analysis code are provided as Supplementary File S6, from which all reported values can be reproduced. Any further data are available from the corresponding author on reasonable request.
