# Supplementary material for: Secondary traumatic stress and psychological distress among responders to the 2023 Kahramanmaraş earthquakes: a systematic review and meta-analysis
Source: Front Public Health. 2026 Oct 8;14:1967067. doi: 10.3389/fpubh.2026.1967067 (PMC13686910; doi:10.3389/fpubh.2026.1967067)
Supplement: Supplementary file 1 [file Table_1.DOCX]

**Supplementary File S1 — Search strategies**

*Secondary traumatic stress and psychological distress among responders to the 2023 Kahramanmaraş earthquakes: a systematic review and meta-analysis*

*PROSPERO CRD420261289019*

**S1.1 Concept blocks**

The strategy combines three blocks with the Boolean operator AND; terms within a block are combined with OR. The blocks are (i) responder population, (ii) index event and (iii) psychological outcome. Parallel strategies were constructed in English and in Turkish.

**S1.2 English-language strategy**

**Population block.** ("rescue worker*" OR "first responder*" OR "search and rescue" OR "urban search and rescue" OR USAR OR firefighter* OR "fire fighter*" OR paramedic* OR "emergency medical technician*" OR "emergency medical team*" OR "disaster responder*" OR "disaster relief" OR "relief worker*" OR "humanitarian worker*" OR "aid worker*" OR volunteer* OR "health care worker*" OR "healthcare professional*" OR nurse* OR physician* OR "social worker*" OR "psychosocial support" OR "mental health professional*" OR "Red Crescent" OR AFAD OR UMKE)

**Event block.** (Kahramanmaras OR Kahramanmaraş OR Pazarcik OR Pazarcık OR Elbistan OR Ekinozu OR Ekinözü OR ((2023) AND earthquake* AND (Turkey OR Turkiye OR Türkiye OR Syria)))

**Outcome block.** ("secondary traumatic stress" OR "secondary trauma*" OR "vicarious trauma*" OR "compassion fatigue" OR "compassion satisfaction" OR PTSD OR "post-traumatic stress" OR "posttraumatic stress" OR "acute stress" OR depress* OR anxiet* OR burnout OR "burn out" OR resilien* OR "post-traumatic growth" OR "posttraumatic growth" OR "psychological distress" OR "moral injury")

**S1.3 Turkish-language strategy**

**Population block.** ("arama kurtarma" OR itfaiyeci* OR AFAD OR UMKE OR Kızılay OR "afet çalışan*" OR "afet müdahale" OR gönüllü* OR "sağlık çalışan*" OR hemşire* OR hekim* OR "psikososyal destek" OR "sosyal hizmet uzman*" OR paramedik OR "112 personel*" OR "ilk müdahale")

**Event block.** ("6 Şubat 2023" OR "Kahramanmaraş deprem*" OR "Pazarcık" OR "Elbistan" OR "Ekinözü" OR "asrın felaketi")

**Outcome block.** ("ikincil travmatik stres" OR "ikincil travma" OR "travma sonrası stres" OR TSSB OR "merhamet yorgunluğu" OR "merhamet doyumu" OR tükenmişlik OR "psikolojik sağlamlık" OR "psikolojik dayanıklılık" OR depresyon OR anksiyete OR kaygı OR "travma sonrası büyüme" OR "üstlenilmiş travma")

**S1.4 Sources searched, fields, execution dates and yields**

The original search was executed on 30 May 2025. Following peer review, the search was rerun and extended on 2 September 2026, with the National Thesis Centre and TR Dizin added as separate sources. Yields below are for the updated search.

| **Source** | **Fields / limits** | **Records** | **Notes** |
| --- | --- | --- | --- |
| Web of Science Core Collection | Topic (TS=) | 186 | Editions SCI-E, SSCI, ESCI |
| PubMed | Title/Abstract; filtered post hoc to ≥ 6 Feb 2023 | 143 | MeSH not used — the event is too recent for reliable indexing |
| Scopus | TITLE-ABS-KEY | 214 | — |
| PsycINFO | Any field | 97 | Captures psychology-specific Turkish output |
| TR Dizin | Title, abstract, keywords | 258 | Turkish national index; added at the updated search |
| DergiPark | Full-text search | 291 | Hosts most Turkish-language health and social science journals |
| YÖK Ulusal Tez Merkezi | Title, keywords | 58 | Postgraduate theses; grey literature; added at the updated search |
| **Total** |  | **1,247** | **389 duplicates removed, leaving 858 records for screening** |

**S1.5 Supplementary search methods**

Backward citation chasing through the reference lists of all included reports and of all reviews identified during screening, including the two syntheses excluded as secondary sources. Forward citation tracking of all included studies via Google Scholar and Web of Science. Hand-search of the DergiPark "Cited By" field for each included Turkish-language report. Contact with corresponding authors where a study reported medians and ranges only, where distributional parameters were missing, or where a response metric could not be established from the published text.

**S1.6 Amendment to the search after peer review**

Reviewer comment prompted re-examination of search sensitivity. Two studies named by a reviewer were traced to sources indexed in TR Dizin and MDPI and had not been captured by the original strategy. Re-execution with the two Turkish sources added, and with the Ekinözü and "ilk müdahale" terms included, raised the yield from 297 to 1,247 records and the included set from 12 to 21 studies. The shortfall was one of source coverage and execution date rather than of search terms: nine of the additional studies would have been retrievable with the original term list had TR Dizin and the National Thesis Centre been searched at the original date.
