# Supplementary material for: Secondary traumatic stress and psychological distress among responders to the 2023 Kahramanmaraş earthquakes: a systematic review and meta-analysis
Source: Front Public Health. 2026 Oct 8;14:1967067. doi: 10.3389/fpubh.2026.1967067 (PMC13686910; doi:10.3389/fpubh.2026.1967067)
Supplement: Supplementary file 2 [file Table_2.DOCX]

**Supplementary File S2 — PRISMA 2020 checklist**

*Secondary traumatic stress and psychological distress among responders to the 2023 Kahramanmaraş earthquakes: a systematic review and meta-analysis*

*PROSPERO CRD420261289019*

Location refers to the section heading in the main manuscript or to the relevant supplementary file. All items are complete.

| **Item** | **Topic** | **Checklist item** | **Location** |
| --- | --- | --- | --- |
| 1 | Title | Identify the report as a systematic review | Title |
| 2 | Abstract | See PRISMA 2020 for Abstracts checklist | Abstract |
| 3 | Rationale | Describe the rationale in the context of existing knowledge | Introduction, paras 2–5 |
| 4 | Objectives | Provide an explicit statement of objectives | Introduction, para 7 |
| 5 | Eligibility criteria | Specify inclusion/exclusion criteria and grouping | Methods, Eligibility criteria |
| 6 | Information sources | Specify all databases, registers, other sources, and dates | Methods, Information sources; S1.4 |
| 7 | Search strategy | Present full search strategies for all sources | S1.2–S1.3 |
| 8 | Selection process | Specify methods, number of reviewers, independence | Methods, Study selection |
| 9 | Data collection process | Specify methods, number of reviewers, independence | Methods, Study selection; S3 |
| 10a | Data items — outcomes | List and define all outcomes sought | Methods, Eligibility criteria, para 2 |
| 10b | Data items — other | List and define all other variables sought | Methods, Study selection; S3 |
| 11 | Risk of bias assessment | Specify methods, tools, number of reviewers | Methods, Risk of bias; S4 |
| 12 | Effect measures | Specify effect measure(s) for each outcome | Methods, Effect measures and synthesis |
| 13a | Synthesis — eligibility | Describe processes to decide which studies are grouped | Methods, Effect measures and synthesis |
| 13b | Synthesis — preparation | Describe methods to prepare data for synthesis | Methods, Study selection, para 3 (transformations) |
| 13c | Synthesis — tabulation | Describe methods to tabulate or visually display results | Results, Tables 1–6; Figures 1–3 |
| 13d | Synthesis — methods | Describe methods used to synthesise results; model, software | Methods, Effect measures and synthesis; S6 |
| 13e | Synthesis — heterogeneity | Describe methods to explore causes of heterogeneity | Methods, Effect measures and synthesis |
| 13f | Synthesis — sensitivity | Describe sensitivity analyses | Methods, Effect measures and synthesis |
| 14 | Reporting bias assessment | Describe methods to assess risk of bias due to missing results | Methods, Effect measures and synthesis (threshold of ten studies not reached) |
| 15 | Certainty assessment | Describe methods to assess certainty in the body of evidence | Methods, Risk of bias; S4.3 |
| 16a | Study selection | Describe results of search and selection; flow diagram | Results, Study selection; Figure 1 |
| 16b | Excluded studies | Cite studies excluded at full text and give reasons | S5 |
| 17 | Study characteristics | Cite each included study and present characteristics | Tables 1 and 5 |
| 18 | Risk of bias in studies | Present assessments for each included study | Results, Risk of bias; S4.1–S4.2 |
| 19 | Results of individual studies | Present summary statistics and effect estimates | Tables 2–4; Figures 2–3 |
| 20a–d | Results of syntheses | Present syntheses, heterogeneity, sensitivity analyses | Results, Secondary traumatic stress; Compassion fatigue; Pooled prevalence |
| 21 | Reporting biases | Present assessments of risk of bias due to missing results | Results, Pooled prevalence (not assessable at k < 10); Limitations |
| 22 | Certainty of evidence | Present assessments of certainty for each outcome | S4.3 |
| 23a–d | Discussion | Interpretation, limitations, implications | Discussion; Limitations; Conclusion |
| 24a–c | Registration and protocol | Registration details; protocol availability; amendments | Methods, Protocol and registration; Deviations from the registered protocol |
| 25 | Support | Describe sources of financial or non-financial support | Declarations, Funding |
| 26 | Competing interests | Declare competing interests | Declarations, Competing interests |
| 27 | Availability of data and code | Report availability of data, code and other materials | Declarations, Data and code availability; S6 |
