# Supplementary material for: Secondary traumatic stress and psychological distress among responders to the 2023 Kahramanmaraş earthquakes: a systematic review and meta-analysis
Source: Front Public Health. 2026 Oct 8;14:1967067. doi: 10.3389/fpubh.2026.1967067 (PMC13686910; doi:10.3389/fpubh.2026.1967067)
Supplement: Supplementary file 3 [file Table_3.DOCX]

**Supplementary File S3 — Data extraction form**

*Secondary traumatic stress and psychological distress among responders to the 2023 Kahramanmaraş earthquakes: a systematic review and meta-analysis*

*PROSPERO CRD420261289019*

Fields extracted for every included study. One reviewer extracted; a second independently verified every numerical entry against the primary source.

| **Field** | **Definition / coding** | **Type** |
| --- | --- | --- |
| study_id | First author and year | Text |
| provinces | Provinces or setting of data collection | Text |
| design | Cross-sectional / cohort / mixed methods / qualitative / quasi-experimental | Categorical |
| sampling | Consecutive / convenience / snowball / online self-selected / total population | Categorical |
| responder_type | 1 = SAR and fire; 2 = medical and EMS; 3 = psychosocial and mental health; 4 = social work; 5 = unaffiliated volunteer; 6 = mixed | Categorical |
| deployment | 1 = deployed into zone from elsewhere; 2 = resident within affected zone; 3 = based outside the zone, treating evacuated casualties; 4 = mixed or unclear | Categorical — KEY MODERATOR |
| deployment_days | Duration of deployment, median or mean | Continuous |
| months_post | Months between 6 February 2023 and the midpoint of data collection | Continuous — KEY MODERATOR |
| n_analysed | Sample size contributing to the outcome | Integer |
| age_mean / age_sd | Mean age and standard deviation | Continuous |
| pct_female | Percentage female | Continuous |
| instrument | Name, version, number of items, response range | Text |
| adaptation | Turkish validation reference used (e.g. Kâhil 2016; Yıldırım et al. 2018) | Text — MODERATOR |
| cutoff | Applied threshold, if any, with its source | Numeric |
| outcome_mean / outcome_sd | Mean and standard deviation for each outcome, on the raw instrument metric | Continuous |
| outcome_median / q1 / q3 | Median and interquartile range where a mean is not reported | Continuous |
| item_mean / item_sd / n_items | Item-level values and item count where totals are not reported | Continuous |
| n_above_cutoff | Number and percentage scoring above threshold | Integer |
| comparator | Presence and nature of a non-exposed comparison group | Text |
| personal_loss | Proportion bereaved, home destroyed, physically injured; and whether the loss was the responder’s own or a relative’s | Continuous + categorical |
| participation_type | Volunteered / institutionally assigned | Categorical |
| transform | none / item_mean_to_total / wan_from_median_iqr / weighted_from_subgroups | Categorical |
| status | verified (read from primary table) / abstract / pending | Categorical |
| consistency_flags | Free-text record of internal inconsistencies found | Text |

**S3.1 Internal consistency verification**

Every extracted numerical value is checked against the source article’s own tables before entry. Four checks are applied. Subgroup counts must sum to the stated total. Reported percentages must reproduce from their implied numerator and denominator. Reported confidence intervals must reconstruct from the point estimate and standard error. Values reported in the abstract and discussion must agree with the corresponding results table. A fifth check was added at the updated extraction: reported means must be locatable on the stated response metric of the instrument, and reported observed ranges must fall within the instrument’s possible range.

Where a discrepancy is found and the primary table is internally coherent, the table value is used and the discrepancy is logged in the consistency_flags field and in S4. Where the discrepancy cannot be resolved, the affected value is excluded from synthesis and recorded in the excluded_values block of extraction.json.

**S3.2 Declared transformations**

Three transformations were applied to render otherwise incomparable values poolable. Each is declared in the transform field of every affected record, is implemented in code rather than by hand, and is identified wherever the resulting value appears in the manuscript. item_mean_to_total multiplies a reported item mean and its standard deviation by the number of items in the scale, and is applicable only to scales with no reverse-scored items and no missing-item adjustment. wan_from_median_iqr estimates a mean and standard deviation from a median and interquartile range using the estimators of Wan et al. (2014). weighted_from_subgroups reconstructs an overall mean and standard deviation from independent subgroup breakdowns, and is accepted only where two or more independent breakdowns of the same sample converge.
