# Supplementary material for: Secondary traumatic stress and psychological distress among responders to the 2023 Kahramanmaraş earthquakes: a systematic review and meta-analysis
Source: Front Public Health. 2026 Oct 8;14:1967067. doi: 10.3389/fpubh.2026.1967067 (PMC13686910; doi:10.3389/fpubh.2026.1967067)
Supplement: Supplementary file 4 [file Table_4.DOCX]

**Supplementary File S4 — Risk of bias assessment**

*Secondary traumatic stress and psychological distress among responders to the 2023 Kahramanmaraş earthquakes: a systematic review and meta-analysis*

*PROSPERO CRD420261289019*

**S4.1 Analytical cross-sectional and cohort studies (JBI)**

Joanna Briggs Institute critical appraisal checklist for analytical cross-sectional studies, applied to all thirteen quantitative studies. Items: 1 inclusion criteria clearly defined; 2 subjects and setting described in detail; 3 exposure measured validly and reliably; 4 objective standard criteria used for measurement of the condition; 5 confounding factors identified; 6 strategies to deal with confounding stated; 7 outcomes measured validly and reliably; 8 appropriate statistical analysis used. Y = yes, P = partial, N = no.

| **Study** | **1** | **2** | **3** | **4** | **5** | **6** | **7** | **8** | **Overall** |
| --- | --- | --- | --- | --- | --- | --- | --- | --- | --- |
| Türk & Kaya (2023) | Y | Y | P | Y | P | N | Y | P | Moderate |
| Bakirci, Sar & Cetin (2024) | Y | Y | Y | Y | N | N | Y | N | Moderate |
| Topkara et al. (2024) | Y | Y | P | Y | P | N | Y | P | Moderate |
| Akdağ et al. (2024) | Y | Y | Y | Y | P | N | Y | Y | Low–moderate |
| Hugelius et al. (2024) | Y | Y | P | N | N | N | N | P | Moderate–high |
| Sevim & Öksüz Poplata (2024) | Y | Y | P | Y | N | N | P | N | Moderate |
| Etçioğlu et al. (2025) | Y | Y | Y | Y | P | N | Y | P | Moderate |
| Sisman, Cerit & Polat (2025) | Y | Y | P | Y | P | N | Y | Y | Low–moderate |
| Koca, Deniz & İnceoğlu (2025) | Y | Y | P | Y | P | N | P | Y | Moderate |
| Onat et al. (2025) | Y | Y | Y | Y | P | N | Y | P | Moderate |
| Taş et al. (2025) | Y | Y | Y | Y | P | N | Y | Y | Low–moderate |
| Kaya & Kara (2026) | Y | Y | Y | Y | P | N | Y | Y | Low–moderate |
| Derya et al. (2026) | Y | Y | Y | Y | P | N | Y | P | Moderate |

Etçioğlu et al. was additionally appraised with the JBI checklist for cohort studies; attrition from 32 to 29 was reported and accounted for, but no strategy for confounding was stated and the omnibus tests were underpowered. Hugelius et al. scores poorly on items 4 and 7 because outcomes were assessed with investigator-designed single items; the study was retained for narrative description only and contributes to no pooled estimate.

**S4.2 Qualitative and mixed-method reports (JBI)**

Joanna Briggs Institute critical appraisal checklist for qualitative research, summarised by domain. All eight reports stated a clear research question and a congruent methodology; none reported researcher reflexivity in full; participant voice was adequately represented in all but one.

| **Study** | **Philosophical congruity** | **Researcher located culturally** | **Participants adequately represented** | **Overall** |
| --- | --- | --- | --- | --- |
| Koçak et al. (2025) | Partial | No | Partial | Moderate |
| Çakaloğlu et al. (2025) | Yes | No | Yes | Low–moderate |
| Çorbacı, Tansel & Alkan (2025) | Yes | Partial | Yes | Low–moderate |
| Celebi Cakiroglu et al. (2025) | Yes | Partial | Yes | Low–moderate |
| Parlak et al. (2025) | Yes | Partial | Yes | Low–moderate |
| Mert & Koksal (2025) | Yes | No | Yes | Moderate |
| Memiş Doğan & Sevinçli Bayram (2025) | Yes | No | Yes | Moderate |
| Altunbek, Ildırım & Atalar (2026) | Partial | Yes | Yes | Moderate |

Altunbek et al. deliberately collected no sociodemographic data, which the authors justify on grounds of participant safety twelve days after the event but which limits contextual interpretation. Koçak et al. embedded free-text qualitative feedback within a structured survey of 683 personnel rather than conducting interviews, so depth is limited; only the qualitative component contributes here.

**S4.3 Data-quality findings from internal consistency verification**

| **Study** | **Findings** |
| --- | --- |
| Bakirci et al. (2024) | PETLDS subgroup medians reported inverted relative to the instrument cut-off; the accompanying figure confirms the correct direction. The "DASS-21 total" row of Table 2 contains values identical to the PETLDS subgroup values and implies a range of 21–72 against the paper’s own stated range of 4–76; group-stratified DASS-21 values excluded, overall mean retained. The Brief Resilience Scale results paragraph duplicates the PCL-5 paragraph verbatim; no BRS subgroup values recoverable. Discussion reports 37.2% above cut-off for depression and 45.5% for anxiety; recomputation from Table 2 yields 43.5%/21.1% and 65.5%/48.9%, and neither reported figure is derivable. Column totals are internally correct (195 and 28 throughout), so table values were used. Two conflicting ethics approvals appear in the same article (2023/166 dated 12 May 2023 in Methods; 2023/218 dated 2 May 2023 in Declarations). Age reported as 33.7 ± 16.4 with a median of 40 and a range of 23–64, which is not internally coherent. |
| Topkara et al. (2024) | The abstract reports the STSS–compassion fatigue correlation as r = −0.739 while Table 3 and the accompanying text both give +0.739 and describe the association as high and positive. Treated as a sign error in the abstract; the table value was extracted. No a priori sample size calculation; a post hoc power analysis is reported. Sampling was haphazard non-probability. |
| Akdağ et al. (2024) | No inconsistencies detected. The authors report transparently that the target sample size was not reached and that regression assumptions were consequently not met, restricting analysis to correlation. Recruitment via an instant-messaging application introduces uncontrolled self-selection. |
| Türk & Kaya (2023) | Sex-stratified secondary trauma means (women 4.47 ± 2.18, n = 141; men 3.03 ± 1.56, n = 108) reweight to 3.845 against a reported overall mean of 3.56, a gap of 0.29 that rounding does not explain; the sex-stratified figures were not extracted. Equivalent recomputations for resilience, burnout by experience and burnout by participation type all fall within 0.10 and are acceptable. The regression paragraph for parenthood duplicates the marital-status paragraph verbatim, carrying the wrong F, t and p and a sign-flipped B; Table 2 is coherent and was used. Subscale item means reconstruct to a CF-SS total of 50.04 against the reported 51.61. All demographic subgroup counts sum correctly to 249, and the reported minimum sample size of 245 reproduces exactly from the stated formula. |
| Sevim & Öksüz Poplata (2024) | Table 1 percentages for marital status and parenthood are transposed: 80 single is 79.2% not 20.8%, and 21 married is 20.8% not 79.2%; the same swap appears for having children. The Table 3 resilience mean of 127.91 (SD 22.32) cannot be reproduced from any subgroup breakdown; the sex split and the age-band split both reweight to 130.67 with subgroup SDs near 16. The secondary traumatic stress mean reproduces exactly at 31.81 from three independent breakdowns and was used. The regression paragraph for having children is a verbatim copy of the marital-status paragraph with the wrong test statistics and a sign-flipped B; Table 4 is coherent and was used. Four STSS items were removed after the authors’ own factor analysis, so the total is not on the 17–85 metric and did not enter the meta-analysis. |
| Onat et al. (2025) | Ways of Coping subscales do not sum to the reported totals: effective (29.46) plus ineffective (14.91) is 44.37 against a reported total of 56.55; the three effective sub-dimensions sum to 30.52 against a reported 29.46. The optimistic and seeking-social-support rows carry identical values to two decimal places including the standard deviation (8.62 ± 2.66), and the optimistic row carries a duplicated ± sign. STSS values are internally coherent (intrusion 11.20 + avoidance 16.21 + arousal 11.54 = 38.95 against a reported total of 38.94) and were used. Only the 159 participants who delivered care to earthquake casualties meet the exposure criterion; the 84 who did not are recorded in S5. |
| Koca et al. (2025) | The Traumatic Stress Symptom Scale is described in the Methods as seventeen items scored 0 to 3 with a total range of 0–51, but Table 4 reports observed maxima of exactly 68 for both sexes, which is seventeen items scored 1 to 4. Table 3 separately reports an observed range of 14–50, contradicting Table 4 on both bounds. The reported mean of 44.04 cannot be located on a defined metric and was excluded from synthesis pending author clarification. All subgroup breakdowns reweight exactly to the reported totals and PETSSS equals TSSS plus Disability throughout, so the internal arithmetic is sound; the defect is in the stated response metric. Psychological hardiness correlates −0.281 with TSSS and −0.270 with Disability but only −0.202 with their composite, which should sit near −0.28. |
| Taş et al. (2025) | The Introduction dates the earthquakes to 6 February 2022. Table 1 gives the Group 1 female proportion as 54.6% where 32 of 62 is 51.6%; every other cell sums and reproduces correctly. The statement that probable PTSD was present in 76.1% of affected health workers "when suggested cut-off score is selected" specifies no cut-off value, and the cited Turkish validation establishes none; this proportion was excluded from the prevalence meta-analysis. All outcome values are reported as medians with interquartile ranges and were converted per Wan et al. (2014). |
| Kaya & Kara (2026) | No overall mean or standard deviation is reported for the primary outcome; the total was reconstructed from five independent subgroup breakdowns which converge to within 0.01 (39.43–39.44, SD 13.69–13.70). Two different ethics records appear in the same article: Method section gives 21 July 2023, no. 2023/202; the ethics statement gives 19 July 2023, no. 2023/218. Every reported effect size recomputes correctly from the reported means and pooled standard deviation; this is otherwise the most internally coherent study in the set. The effect size for the eight-to-fourteen-day contrast is labelled "medium" at η² = 0.032, which is small by convention. |
| Derya et al. (2026) | Departmental breakdowns overshoot the reported totals for both principal scales: PETLDS reweights to 52.64 against a reported 50.75, and CF-SS to 63.58 against a reported 60.12. Every other subgroup breakdown — sex, occupation, marital status, parental status, and all nine earthquake-experience contrasts — reconciles to the reported totals exactly, and the CF-SS subscales sum correctly (21.26 + 38.85 = 60.11). The totals were used and the departmental rows were not extracted. The sample-size paragraph repeats one sentence four times verbatim, and the Strengths and Limitations section names instruments ("Patient Empowerment Trait Scale for Depression Scale", "Contextual Stress Scale") that are not those used in the study; the authors acknowledge use of a generative language tool for formatting. Data were collected in November–December 2024 but participants were asked to report on the first 72 hours after the earthquake, a recall interval of approximately 22 months. |
| Memiş Doğan & Sevinçli Bayram (2025) | Two different ethics approval numbers appear in the same article: E.3668 in the Methods, and E-84771431-050.01.04-91196 dated 11 May 2023 in the ethics statement. All demographic cells in Table 1 reconcile with the text. |
| Altunbek, Ildırım & Atalar (2026) | The abstract states ten tent cities; the Methods state fourteen tent cities and one guesthouse; Table 1 lists fourteen sites of which one is the guesthouse. The Methods state ten provinces, but the ten rows of Table 1 collapse to seven provinces once the paired Adıyaman, Hatay and Gaziantep entries are combined; the province count was taken from Table 1. The ethics committee is redacted in the version obtained, though the decision number and date are given and correctly precede data collection. Responder and survivor counts sum correctly to 39 and 66. |

**S4.4 Pattern across the evidence base**

Three features recur often enough to merit comment rather than itemisation. Conflicting ethics approval details appear within a single article in three studies. Verbatim duplication of a results paragraph under a different heading, carrying the wrong statistics, appears in three studies. And in four studies a reported value could not be located on the instrument’s stated response metric. None of these is individually disqualifying and all were handled by preferring the internally coherent primary table, but together they bear on the confidence that can be placed in unverified values from this literature and are reflected in the statistical analysis domain of the appraisals above.

**S4.5 Certainty of the body of evidence**

Certainty was appraised narratively rather than with GRADE, since no outcome was assessed in an interventional design. Certainty in the pooled Secondary Traumatic Stress Scale total is low: four studies, all non-probability samples, extreme unexplained heterogeneity in the unstratified analysis. Certainty in the exposure subgroup contrast is moderate: it is corroborated between studies, within a study by design, and within samples as a dose gradient, but rests on unadjusted comparison. Certainty in the pooled compassion fatigue total is low: three studies, one requiring rescaling. Certainty in the pooled probable PTSD prevalence is low to very low: three studies, three different instrument–threshold combinations, no affected-resident sample contributing, and a between-study variance estimate of exactly zero that three studies cannot support.

**S4.6 Note on instrument adaptation**

Studies contributing to the primary outcome used two Turkish adaptations of the Secondary Traumatic Stress Scale: Kâhil (2016) in Topkara et al. and Kaya and Kara, and Yıldırım et al. (2018) in Akdağ et al., Onat et al. and Taş et al. Both derive from the same 17-item Bride et al. (2004) instrument with identical 1–5 response scaling and a 17–85 range, so the raw metric is comparable. Adaptation was examined as a pre-specified moderator and does not separate: the Kâhil studies returned 39.43 and 55.60 and the Yıldırım studies 40.55, 41.73 and 42.00.
