# Supplementary material for: Secondary traumatic stress and psychological distress among responders to the 2023 Kahramanmaraş earthquakes: a systematic review and meta-analysis
Source: Front Public Health. 2026 Oct 8;14:1967067. doi: 10.3389/fpubh.2026.1967067 (PMC13686910; doi:10.3389/fpubh.2026.1967067)
Supplement: Supplementary file 5 [file Table_5.DOCX]

**Supplementary File S5 — Studies excluded at full-text assessment, and reports not retrieved**

*Secondary traumatic stress and psychological distress among responders to the 2023 Kahramanmaraş earthquakes: a systematic review and meta-analysis*

*PROSPERO CRD420261289019*

**S5.1 Excluded at full text (n = 10)**

| **Reference** | **Reason** | **Detail** |
| --- | --- | --- |
| Bilge, M., & Zafer, M. (2026). OPUS – Journal of Society Research, 23, e1922409. | Population–exposure mismatch | 497 İstanbul fire department personnel surveyed on general occupational trauma exposure. The 2023 earthquakes are not the index exposure and no deployment variable is recorded. Additional concerns noted during assessment: the abstract states a positive STS–social support association while all reported correlations are negative; the demographic table has n and % transposed; gender counts sum to 494 against a stated N of 497; Figure 1 path coefficients conflict with Table 6; reported 95% CIs are not reconstructible from B ± 1.96·SE. |
| Gökçen, O., Karataş, K. S., Akkuş, M., et al. (2024). Turkish Journal of Psychiatry, 35(4), 263. | Population–exposure mismatch | Secondary traumatic stress symptoms in social media users following the index earthquakes. Exposure is via media only, which the protocol excludes explicitly. |
| Özkan Bardakcı, M., Kaya, Ç. S., & Bardakcı, Ş. (2025). Disaster Medicine and Public Health Preparedness, 19, e169. | Population–exposure mismatch | Post-earthquake trauma levels among individuals affected by the earthquakes. Participants are survivors, not personnel acting in a response role. |
| Grossi, E., Marocco, S., Cortini, M., Galanti, T., & Talamo, A. (2025). Social Sciences, 14(12), 708. | Not a primary study | Rapid systematic review with narrative synthesis, all natural hazard types worldwide. Cited and differentiated in the Introduction; contributes no primary data. |
| Sehlikoğlu, Ş., Yilmaz Karaman, I. G., Yastıbaş Kaçar, C., & Canakci, M. E. (2023). Klinik Psikiyatri Dergisi, 26(4), 309–318. | Not a primary study | Systematic review of earthquake and healthcare worker mental health. Search predates the index event. Cited in the Introduction. |
| Ruhm, F., Çalıkoğlu, E. O., Karadağ, M., et al. (2026). BMC Public Health, 26, 1830. | Not a primary study | Scoping review of post-earthquake burnout among doctors and nurses in Türkiye and Syria, 1990–2024. Cited and differentiated in the Introduction; also the source of the Ministry of Health personnel figures reported there. |
| Patwary, M. M., Ashraf, S., Swed, S., Beaglehole, B., & Shoib, S. (2023). Annals of Work Exposures and Health, 67(8), 1018–1021. | Not a primary study | Letter to the Editor with no primary data. Cited in the Introduction as the source of the Özen and Sir (2004) historical comparator. |
| Kalkan, A., & Gerdan, S. (2025). Afet ve Risk Dergisi, 8(3), 667–680. | Outcome not measured with an established instrument | Nineteen search-and-rescue personnel who travelled from Kocaeli to Hatay. Occupational health and safety risk assessment using an L-type 5×5 matrix; psychological and physical fatigue appears as a scored risk item, not as a measured construct. Cited in the Introduction as the source of the search-and-rescue personnel figure. |
| Öztürk, M. H., & Kuday, A. D. (2025). Journal of Health Psychology, 30(12), 3246–3259. | Outcome not among those specified | 275 search-and-rescue workers. Psychometric validation of the Turkish Work-Related Stress Scale; the outcome is occupational stress, which is not among the primary or secondary outcomes listed in the protocol. Cited in the Introduction. |
| Akçay, E., Çöp, E., Şenses Dinç, G., et al. (2025). American Journal of Orthopsychiatry, 95(2), 212–222. | Overlapping sample | 71 doctors and 92 nurses at the same Ankara children’s hospital, over a period overlapping that of Onat et al. (2025). Participant overlap could not be excluded from the published reports, and the two could not be included together without risk of double-counting. Onat et al. was retained because it reports the primary outcome on the pooled metric. |

**S5.2 Partial exclusion within an included study**

Onat et al. (2025) enrolled 243 health workers at a hospital outside the affected provinces. Only the 159 who delivered clinical care to evacuated earthquake casualties meet the exposure criterion in §2.2; the 84 who did not are excluded and are treated in the source article as a comparison group. The subgroup contrast between them is reported in Table 3 of the manuscript. Only the 159 eligible participants enter the sample total, Table 2 and the pooled analyses. The source article reports the relatives’ exposure contrasts, the treated-versus-untreated contrast and the correlations only for its full sample of 243; these values therefore include the 84 excluded staff, are labelled as full-sample values in Tables 3 and 4 of the manuscript, are presented descriptively and enter no pooled estimate. The source reports sex composition (74.1% female) for the full sample only.

**S5.3 Reports sought but not retrieved (n = 3)**

Three reports were identified as potentially eligible and were sought at full text, but could not be obtained within the review period despite institutional requests and direct contact with the corresponding authors. They are recorded here so that a future update can begin from them.

| **Reference** | **Note** |
| --- | --- |
| Polat, H., et al. (2025). Disaster Medicine and Public Health Preparedness, 19, e243. https://doi.org/10.1017/dmp.2025.10153 | Reported in the indexed abstract as 313 health care workers assessed with the Brief Psychological Resilience Scale and the Traumatic Stress Symptom Scale-5, of whom 39.6% met criteria for post-traumatic stress disorder. Had this proportion proved extractable it would probably have moved the pooled prevalence estimate materially, and its absence is the principal reason that estimate is described as provisional in the manuscript. |
| Tosun, C. (2025). Journal of Interpersonal Violence. Advance online publication. https://doi.org/10.1177/08862605251355975 | Secondary traumatic stress among mental health professionals working with people affected by the index earthquakes. Reports the primary outcome; would probably have contributed to the pooled analysis. |
| Yılmaz, T., & Bekaroğlu, E. (2025). Professional Psychology: Research and Practice, 56(6), 505–515. https://doi.org/10.1037/pro0000651 | Factors related to secondary traumatic stress among psychologists following the index earthquakes. Reports the primary outcome; would probably have contributed to the pooled analysis. |

The effect of these three omissions is discussed in the Limitations section of the manuscript. All three are cited in the Introduction as part of the event-specific literature.
