# Supplementary material for: Secondary traumatic stress and psychological distress among responders to the 2023 Kahramanmaraş earthquakes: a systematic review and meta-analysis
Source: Front Public Health. 2026 Oct 8;14:1967067. doi: 10.3389/fpubh.2026.1967067 (PMC13686910; doi:10.3389/fpubh.2026.1967067)
Supplement: Supplementary file 6 [file Table_6.DOCX]

**Supplementary File S6 — Analysis code and data**

*Secondary traumatic stress and psychological distress among responders to the 2023 Kahramanmaraş earthquakes: a systematic review and meta-analysis*

*PROSPERO CRD420261289019*

The extraction dataset is provided as extraction.json and the analysis pipeline as meta_pipeline.py. Running the pipeline against the dataset reproduces every pooled value reported in the manuscript. The pipeline requires only NumPy and SciPy.

**S6.1 What the pipeline implements**

Restricted maximum likelihood estimation of between-study variance (τ²). Random-effects pooling with Hartung–Knapp–Sidik–Jonkman adjusted confidence intervals. Freeman–Tukey double arcsine transformation for prevalence, back-transformed at the harmonic mean sample size. Cochran’s Q, I² and 95% prediction intervals. Wilson score intervals for individual proportions. Hedges g with the small-sample correction. The three declared data transformations described in S3.2. And a protocol guard that refuses to return any pooled estimate below the pre-specified minimum of three contributing studies, raising an error rather than reporting a value.

Each record in extraction.json carries a status field taking the values verified, abstract or pending. Only records marked verified are admitted to any analysis, and the distinction is enforced in code rather than by convention. Records whose values were excluded during verification are retained in an excluded_values block with the reason for exclusion, so that the decisions documented in S4.3 can be audited against the data.

**S6.2 Pipeline output**

Verbatim output of python meta_pipeline.py run against extraction.json on 10 September 2026:

```
==========================================================================
PRIMARY OUTCOME  Secondary Traumatic Stress Scale total (17-85)
   Topkara et al. (2024)              n= 475   55.60 +/- 12.30
   Akdag et al. (2024)                n=  41   41.73 +/- 11.11
   Onat et al. (2025)                 n= 159   40.55 +/- 13.10
   Kaya & Kara (2026)                 n= 341   39.43 +/- 13.70
  k = 4
  Pooled = 44.38  95% CI 32.25 to 56.50
  tau^2 = 57.53   Q(3) = 379.2, p = 0   I^2 = 99.2%
  95% prediction interval: 17.36 to 71.39
  Fixed-effect pooled = 47.89

SENSITIVITY  all six exposure groups
  k = 6
  Pooled = 43.35  95% CI 35.17 to 51.53
  tau^2 = 58.38   Q(5) = 412.4, p = 0   I^2 = 98.8%
  95% prediction interval: 22.07 to 64.63
  Fixed-effect pooled = 47.58

SUBGROUP  resident in / affected by the disaster zone
   Topkara et al. (2024)              n= 475   55.60 +/- 12.30
   Akdag et al. (2024)                n=  41   41.73 +/- 11.11
   Tas et al. (2025), affected        n=  71   48.67 +/- 15.13
  k = 3
  Pooled = 48.79  95% CI 31.48 to 66.11
  tau^2 = 46.96   Q(2) = 66.7, p = 3.22e-15   I^2 = 97.0%
  95% prediction interval: 14.60 to 82.99
  Fixed-effect pooled = 53.81

SUBGROUP  deployed from elsewhere, or receiving evacuees
   Onat et al. (2025)                 n= 159   40.55 +/- 13.10
   Kaya & Kara (2026)                 n= 341   39.43 +/- 13.70
   Tas et al. (2025), deployed        n=  62   33.33 +/- 19.73
  k = 3
  Pooled = 38.40  95% CI 29.68 to 47.12
  tau^2 = 8.21   Q(2) = 7.1, p = 0.029   I^2 = 71.7%
  95% prediction interval: 23.30 to 53.50
  Fixed-effect pooled = 39.46

  Subgroup difference (affected - deployed) = 10.40 points
  approximate 95% CI 1.56 to 19.23

==========================================================================
SECONDARY OUTCOME  Compassion Fatigue Short Scale total (13-130)
   Topkara et al. (2024)              n= 475   71.70 +/- 28.30
   Derya et al. (2026)                n= 183   60.12 +/- 25.95
   Turk & Kaya (2023)                 n= 249   51.61 +/- 24.18
  k = 3
  Pooled = 61.17  95% CI 36.05 to 86.29
  tau^2 = 100.27   Q(2) = 102.2, p = 0   I^2 = 98.0%
  95% prediction interval: 11.30 to 111.04
  Fixed-effect pooled = 62.63

==========================================================================
SECONDARY OUTCOME  Probable PTSD prevalence
   Bakirci et al. (2024)               36/223  =  16.1%  [11.9-21.5]  (PCL-5 >= 41 (Morrison et al., 2021))
   Etcioglu et al. (2025)               4/32   =  12.5%  [5.0-28.1]  (PCL-5 >= 47 (Boysan et al., 2017), 2 weeks)
   Akdag et al. (2024)                  4/41   =   9.8%  [3.9-22.5]  (PTSD-SS >= 24 (Evren et al., 2016))
  k = 3   total events 44/296
  Pooled prevalence = 14.5%  95% CI 8.5 to 21.5
  tau^2 = 0.000   Q(2) = 1.02, p = 0.599   I^2 = 0.0%
  95% prediction interval: 8.5 to 21.5

==========================================================================
EXPOSURE CONTRASTS  Hedges g computed from reported subgroup means
   Topkara (2024)         secondary traumatic stress physical injury to responder     g =  0.99  [ 0.70,  1.29]
   Topkara (2024)         secondary traumatic stress home destroyed                   g =  0.70  [ 0.42,  0.97]
   Topkara (2024)         secondary traumatic stress bereavement                      g =  0.41  [ 0.23,  0.60]
   Topkara (2024)         compassion fatigue         physical injury to responder     g =  0.80  [ 0.51,  1.09]
   Topkara (2024)         compassion fatigue         home destroyed                   g =  0.49  [ 0.21,  0.76]
   Onat (2025)            secondary traumatic stress relative died                    g =  0.62  [ 0.06,  1.18]
   Onat (2025)            secondary traumatic stress relative injured                 g =  0.40  [-0.07,  0.87]
   Onat (2025)            secondary traumatic stress relative financial/shelter loss  g =  0.16  [-0.11,  0.43]
   Onat (2025)            secondary traumatic stress treated earthquake casualties    g =  0.36  [ 0.09,  0.62]
   Kaya & Kara (2026)     secondary traumatic stress assigned rather than volunteered g =  0.47  [ 0.25,  0.69]
   Kaya & Kara (2026)     secondary traumatic stress personal trauma history          g =  0.30  [ 0.09,  0.52]

==========================================================================
BELOW POOLING THRESHOLD (reported narratively, not as results)
   cfss_secondary_trauma_subscale: k = 2  -> guard refuses to pool
   petlds_total: k = 2  -> guard refuses to pool
   depression_above_threshold: k = 2  -> guard refuses to pool
```

**S6.3 Notes on two outputs**

The prediction interval for pooled prevalence coincides with the confidence interval because τ² is estimated at exactly zero. This is the correct behaviour of the estimator and not an error: with three studies, an estimate of zero between-study variance should be read as absence of evidence of heterogeneity rather than as evidence of consistency, and the coincidence of the two intervals is itself a signal that the estimate is fragile. This is stated in the manuscript.

Three outcomes are listed as falling below the pooling threshold and the guard refuses to pool them. Their values are retained in the dataset so that a future update, with additional studies, can pool them without re-extraction.

**S6.4 File manifest**

| **File** | **Contents** |
| --- | --- |
| extraction.json | Full extraction dataset: primary outcome records, compassion fatigue records, prevalence records, exposure contrasts, below-threshold outcomes and excluded values, each with status and transformation fields |
| meta_pipeline.py | Analysis pipeline. Run as python meta_pipeline.py for the output reproduced above, or python meta_pipeline.py --json for machine-readable results |
